# Discovery of a polymer resistant to bacterial biofilm, swarming, and encrustation

**DOI:** 10.1126/sciadv.add7474

**Published:** 2023-01-25

**Authors:** Jean-Frédéric Dubern, Andrew L. Hook, Alessandro M. Carabelli, Chien-Yi Chang, Christopher A. Lewis-Lloyd, Jeni C. Luckett, Laurence Burroughs, Adam A. Dundas, David J. Humes, Derek J. Irvine, Morgan R. Alexander, Paul Williams

**Affiliations:** ^1^ National Biofilms Innovation Centre, University of Nottingham Biodiscovery Institute, School of Life Sciences, University of Nottingham, University Park, Nottingham NG7 2RD, UK.; ^2^ Advanced Materials and Healthcare Technologies, School of Pharmacy, University of Nottingham, University Park, Nottingham NG7 2RD, UK.; ^3^ Division of Gastrointestinal Surgery, Nottingham Digestive Diseases Centre NIHR Biomedical Research Unit, University of Nottingham and Nottingham University Hospitals NHS Trust, School of Medicine, Queen’s Medical Centre, Nottingham NG7 2UH, UK.; ^4^ Centre for Additive Manufacturing, Faculty of Engineering, University of Nottingham, University Park, Nottingham NG7 2RD, UK.

## Abstract

Innovative approaches to prevent catheter-associated urinary tract infections (CAUTIs) are urgently required. Here, we describe the discovery of an acrylate copolymer capable of resisting single- and multispecies bacterial biofilm formation, swarming, encrustation, and host protein deposition, which are major challenges associated with preventing CAUTIs. After screening ~400 acrylate polymers, poly(*tert*-butyl cyclohexyl acrylate) was selected for its biofilm- and encrustation-resistant properties. When combined with the swarming inhibitory poly(2-hydroxy-3-phenoxypropyl acrylate), the copolymer retained the bioinstructive properties of the respective homopolymers when challenged with *Proteus mirabilis*, *Pseudomonas aeruginosa*, *Staphylococcus aureus*, and *Escherichia coli*. Urinary tract catheterization causes the release of host proteins that are exploited by pathogens to colonize catheters. After preconditioning the copolymer with urine collected from patients before and after catheterization, reduced host fibrinogen deposition was observed, and resistance to diverse uropathogens was maintained. These data highlight the potential of the copolymer as a urinary catheter coating for preventing CAUTIs.

## Teaser

A bioinstructive copolymer inhibits bacterial biofilm formation, swarming and encrustation.

## INTRODUCTION

Indwelling urinary tract catheters are the most commonly used prosthetic medical devices with some 15 to 25% of patients requiring bladder catheterization during hospitalization (*1*). However, they promote catheter-associated urinary tract infections (CAUTIs), which are responsible for some 75 to 80% of hospital-acquired urinary tract infections occurring annually worldwide (*2*–*4*). Taking into account the additional costs attributed to patients admitted to intensive care units, CAUTIs carry an annual economic burden estimated at $1.7 billion in the United States alone (*2*). Historically, *Proteus mirabilis* has been the predominant cause of CAUTIs in chronically catheterized individuals (*5*), but other common pathogens include the Gram negatives, *Escherichia coli*, *Pseudomonas aeruginosa*, *Enterobacter*, and *Klebsiella*, as well as Gram positives including enterococci and staphylococci (*6*). Long-term catheterization usually results in multispecies infections (*7*, *8*).

Despite intensive research, the discovery of biomaterials for urinary tract catheters capable of preventing biofilm-centered infections has proved extremely difficult (*9*). Efforts to develop biocompatible catheter materials that resist biofilm formation have focused on the incorporation of antimicrobial agents into the biomaterial (*9*, *10*). However, silver-coated catheters have been disappointing in clinical use, while impregnation with antimicrobials increases the challenges associated with the emergence of multiantibiotic resistance (*11*, *12*). CAUTI prevention is further complicated by bacterial urease production. While *P. mirabilis* is the most potent producer, *Ps. aeruginosa* and *Staphylococcus aureus* strains also express ureases. These hydrolyze urea and increase urinary pH (*13*), leading to device encrustation (biomineralization) by crystalline deposits that can result in catheter blockage and the formation of “infection stones” in the bladder and kidneys, causing irreversible renal damage and an increased rate of morbidity and mortality (*14*, *15*). A laboratory study of 18 different silicone and latex catheters, some with hydrogel or silver coatings (*16*), showed that none resisted *Proteus*-driven encrustation. In addition, *P. mirabilis* is able to differentiate into hyperflagellated cells enabling movement along the catheter surface via a coordinated motility mode known as swarming migration (*6*, *15*). In a simple catheter bridge assay, *P. mirabilis* swarmed over silicone, hydrogel, and silver/hydrogel surfaces more effectively than any of the other common urinary tract pathogens tested (*17*). Furthermore, urinary tract catheterization induces an inflammatory response releasing proteins such as fibrinogen (Fg) that accumulate on silicone catheter surfaces (*18*, *19*). Uropathogens such as *Enterococcus faecalis* and *S. aureus* have Fg-binding proteins that facilitate colonization and biofilm formation on Fg-coated surfaces and potentiate infection (*18*, *19*). Consequently, the ideal urinary catheter biomaterial should offer intrinsic resistance to bacterial biofilm formation, swarming, encrustation, and host protein deposition.

Traditional strategies for discovering promising polymeric biomaterials for medical device applications generally involve time-consuming, iterative investigations of individual polymers that can be further optimized through surface structure-property relationships. For example, polymer brush coatings and zwitterionic polymers (*9*) that reduce biofilm formation have been described and offer potential advantages over silicone. Alternatively, high-throughput approaches based on microarray platforms offer opportunities for rapid parallel screening of polymer libraries (*20*). In previous studies, more than 20,000 assays on 1300 unique copolymers were used to correlate multispecies bacterial attachment with surface chemistry using multivariate surface time-of-flight secondary ion mass spectrometry (TOF-SIMS) (*21*, *22*). This facilitated the discovery of a class of acrylate polymers that resisted bacterial biofilm formation in vitro and in vivo (*21*, *22*). In this case, the most effective polymer chemistries were formed from weakly amphiphilic acrylates with rigid hydrocarbon pendant groups (*23*, *24*), a finding that could not have been predicted from our current understanding of bacterial surface interactions.

Here, we describe the application of a microarray screen to identify polymers capable of resisting *Proteus* biofilm formation, inhibiting swarming motility, and reducing biomineralization that retained these bioinstructive properties in both single- and mixed-species assays with other CAUTI pathogens. We also show that when preconditioned with urine collected from patients before and after catheterization and compared with silicone, the copolymer resists host Fg deposition that is associated with enhanced biofilm formation by pathogens that express Fg receptors.

## RESULTS

### Microarray screen for polymers resistant to *P. mirabilis* biofilm formation

**Fig. 1.**
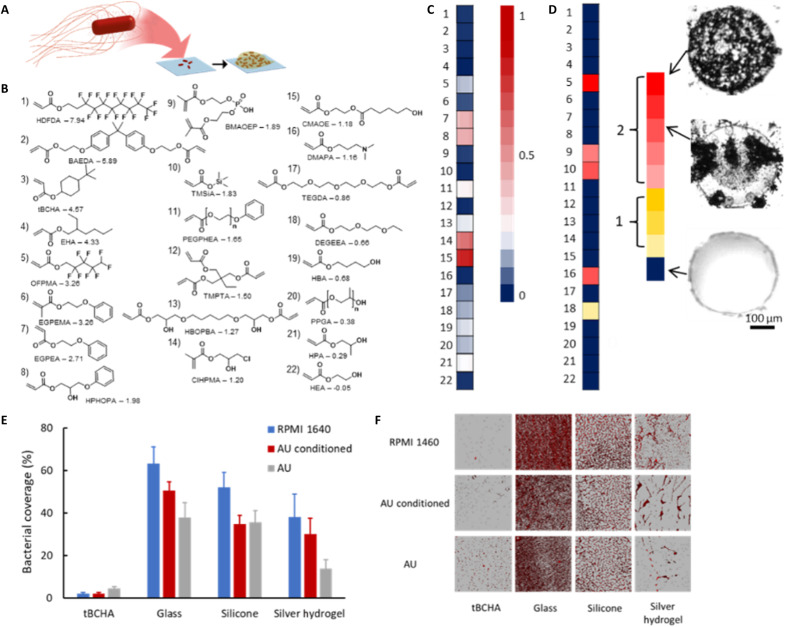
Microarray screen for acrylate copolymers resistant to *Proteus* biofilm formation and biomineralization. (**A**) Schematic depiction of the *Proteus* biofilm assay. (**B**) Chemical structures of the monomers used with their associated clogP values and monomer acronyms (full chemical names are given in Supplementary Materials and Methods). (**C**) Intensity scale plot for fluorescence intensity (*F*_PM_) obtained after incubation of *dsRed*-tagged *P. mirabilis* for 72 hours with the copolymer microarray. Values are indicated by the intensity scale on the right. For each sample, the center of the associated square is colored according to the mean value (*n* = 3), while the left and right portions are respectively colored ± SD. (**D**) Biomineralization susceptibility screen. The extent of biomineralization after a 24-hour incubation in AU of each homopolymer [labels according to (B)] according to the relative scale (from 0 to 1). Bright-field images of examples of polymer spots with high and low biomineralization are shown. Each image is 400 μm × 400 μm. (**E**) *Proteus* biofilm surface coverage after a 72-hour incubation on glass, silicone-, silver hydrogel–, or tBCHA-coated silicone catheter segments in RPMI 1640 (blue), in RPMI 1640 on AU-conditioned (by incubation for 72 hours in AU) polymer coupons (red), or in AU (gray). Error bars are equal to ± 1 SD for three independent replicates. (**F**) Corresponding confocal microscopy images for (E) of *dsRed*-tagged *Proteus* growing on each surface. Each image is 160 μm × 160 μm.

To identify materials capable of preventing *Proteus* biofilm formation (Fig. 1), a microarray composed of 496 polymers as 300-μm-diameter spots on a glass slide was prepared. The core group of 16 homopolymers (numbered 1 to 16; Fig. 1B) was selected to cover a broad chemical space, to exhibit a range of calculated log partition coefficient (clogP) values from −0.05 to 7.94, and to include molecular moieties previously shown to influence bacterial biofilm formation (fluorocarbon, benzyl, cyclic hydrocarbon, hydroxyl, and ethylene glycol groups) (*22*, *25*). To test the effect of copolymerization, the core group of monomers was then deposited in combination with six additional monomers that had previously been shown to induce high, medium, or low bacterial biofilm formation (*22*) at concentrations of 10, 30, 40, 50, and 100% (v/v). The microarray slides were incubated with *dsRed*-tagged *P. mirabilis* 1885 for 72 hours in RPMI 1640; Fig. 1C shows a range of fluorescent values (the highest levels are in red and the lowest in blue) providing a quantitative measure (*F*_PM_) of *P. mirabilis* biofilm formation on each polymer.

Considering first the homopolymers, on 8 of the 16, low levels of biofilm formation were observed (fig. S1A). The low *P. mirabilis* fluorescence *F*_PM_ value for poly(*tert-*butyl cyclohexyl acrylate) [poly(tBCHA)] was consistent with the low biofilm levels previously observed on this polymer using *Ps. aeruginosa*, *S. aureus*, and *E. coli* (*22*). In contrast, a low *F*_PM_ value was observed on poly(hydroxyethyl acrylate) (polyHEA), although for *Ps. aeruginosa*, *S. aureus*, and *E. coli*, high biofilm formation was observed for this material (*21*, *22*). Although the four monomers with the highest clogP values (Fig. 1B) exhibited low biofilm formation, no overall correlation between *F*_PM_ and clogP was observed. For example, the materials with the highest and lowest clogP values, poly(heptadecafluorodecyl acrylate) [poly(HDFDA)] and poly(HEA), respectively (Fig. 1B), exhibited comparably low biofilm levels (Fig. 1C and fig. S1A), demonstrating that considering monomer hydrophobicity in isolation is a poor predictor of biofilm prevention (*26*).

A key requirement for selecting a lead biofilm-resistant monomer for further development was the potential to add a comonomer that would provide additional functionality, such as preventing swarming, without compromising biofilm resistance. The addition of the comonomers reduced the biofilm resistance of the eight best homopolymers. The addition of tBCHA, bisphenol A ethoxylate diacrylate (BAEDA), and HDFDA all resulted in low to medium biofilm formation when copolymerized with the high bacterial biofilm supporting test monomers hydroxy-3-phenoxypropyl acrylate (HPhOPA) or caprolactone 2-(methacryloyloxy)ethyl ester (CMAOE), up to the addition of 40% (v/v) of the test monomer (Fig. 1B and fig. S1B). To determine whether any of the candidate homopolymers promoted biomineralization in the absence of bacteria, a microarray was incubated in artificial urine (AU) and assessed for the presence of opaque crystals using bright-field light microscopy. Substantial biomineralization was observed on the homopolymer of trimethylsilyl acrylate (TMSiA) (Fig. 1D), indicating that it was unsuitable for further evaluation despite its low *F*_PM_ value after incubation with *P. mirabilis* in RPMI medium. Poly(tBCHA) was selected as the lead monomer for further investigation as it displayed no biomineralization, had previously shown resistance to a broad range of bacterial species, and was an inexpensive monoacrylate (*22*, *27*).

To determine whether poly(tBCHA) maintained biofilm inhibitory properties upon scale-up, 10-mm-diameter polymer-coated glass coverslips were prepared by ultraviolet (UV) curing of a dip-coated film of monomer and photoinitiator and assessed for biofilm formation. Borosilicate glass as well as silicone catheter and silver hydrogel–coated catheter segments were used as reference materials. *P. mirabilis* biofilm formation was quantified after incubation for 72 hours at 37°C in RPMI or RPMI following surface conditioning by incubating in AU or after growth in AU (Fig. 1, E and F). For all three conditions, *P. mirabilis* biofilm surface coverage on poly(tBCHA) (100:0) was <5% in contrast to glass, silicone, and silver hydrogel where coverage was ~15 to 65% depending on the surface and growth medium (Fig. 1, E and F). Incubation of *P. mirabilis* with poly(tBCHA) showed no evidence of bacterial planktonic growth inhibition (fig. S2A).

### Discovery of polymers that prevent swarming migration

**Fig. 2.**
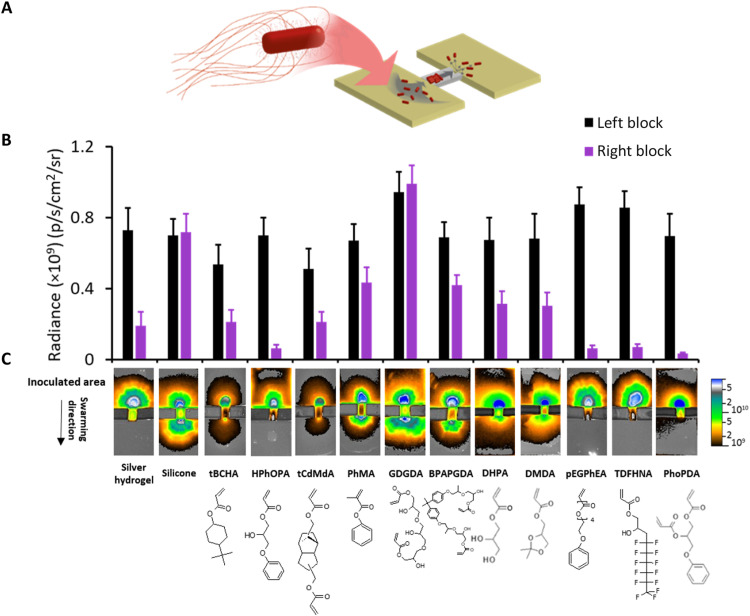
Swarming of *Proteus* over polymer-coated catheter segments. (**A**) Schematic depiction of the bridge swarming assay. (**B**) *dsRed*-tagged *Proteus* was inoculated on one side of the bridge linking two unconnected LB agar blocks and the fluorescence intensity (as radiance) on the lower agar block quantified via fluorescence imaging after incubation for 16 hours. Error bars are equal to ± 1 SD for at least three independent replicates. (**C**) Top: Fluorescence images of the catheter bridge assays. Bottom: Monomer structures corresponding to each fluorescence image. Monomers were tricyclodecane-dimethanol diacrylate (tCdMdA), phenyl methacrylate (PhMA), glycerol 1,3-diglycerolate diacrylate (GDGDA), bisphenol A propoxylate glycerolate diacrylate (BPAPGDA), dihydroxypropyl acrylate (DHPA), 2,2-dimethyl dioxolan-4-yl methyl acrylate (DMDA), tridecafluoro-2-hydroxynonyl acrylate (TDFHNA), and phenoxypropyl diacrylate (PhoPDA).

Unlike most bacteria, *P. mirabilis* can swarm over harder surfaces (e.g., silicone rubber) (*28*) and so migrate along urinary catheters (*29*). We therefore investigated whether poly(tBCHA) inhibited *P. mirabilis* swarming as well as biofilm formation. Silicone catheter segments were coated with poly(tBCHA), and *P. mirabilis* swarming was assessed using a catheter bridge model (*17*). By inoculating fluorescently labeled *P. mirabilis* on one side of a bridge linking two otherwise unconnected LB agar blocks (Fig. 2A and fig. S3), a quantitative indication of migration was achieved by comparing the surface fluorescence (radiance; photons/sec/cm^2^/steradian) on the agar blocks (Fig. 2). Under these conditions, *P. mirabilis* migrated across the uncoated silicone but not across the silver hydrogel–coated catheter. Despite resisting biofilm formation, poly(tBCHA) did not prevent *P. mirabilis* swarming across the poly(tBCHA) (Fig. 2). However, poly(hydroxy-3-phenoxypropyl acrylate) [poly(HPhOPA)] was found to be resistant to swarming (Fig. 2), despite this polymer not preventing biofilm formation (Fig. 1C).

We therefore chose to explore the antiswarming properties of polymers structurally similar to poly(HPhOPA), phenyl and hydroxyl functionality containing/omitting, and replacement of the phenyl/hydroxyl groups with hydrophobic/hydrophilic substitutions. Polymers produced from monomers lacking either the hydroxyl [phenyl methacrylate (PhMA)] or phenyl moieties [dihydroxypropyl acrylate (DHPA), 2,2-dimethyl dioxolan-4-yl methyl acrylate (DMDA), and glycerol 1,3-diglycerolate diacrylate (GDGDA)] failed to prevent swarming as did the polymer of the diacrylate bisphenol A propoxylate glycerolate diacrylate (BPAPGDA) that contained both (Fig. 2). Three polymers prepared from poly(ethylene glycol) phenyl ether acrylate (PEGPHEA), tridecafluoro-2-hydroxynonyl acrylate (TDFHNA), and phenoxypropyl diacrylate (PhoPDA) inhibited swarming (Fig. 2). In each case, the monomers contained both hydrophobic and hydrophilic moieties within their pendant groups. TOF-SIMS analysis (*30*) of the PEGPHEA and PhoPDA polymers confirmed the presence and distribution of benzyl pendant groups at the polymer surface (fig. S4 and table S1). To increase the physiological relevance of these experiments, the coated catheter segments were incubated in AU, and the bridge assays were repeated with similar results (fig. S5).

### Correlation of swarming motility with monomer molecular descriptors

To explore monomer (and so polymer) structure-function relationships in the context of swarming inhibition, a partial least squares (PLS) regression was performed using molecular descriptors. Those that prevented swarming were assigned a value of 1, while those unable to prevent swarming were assigned a value of 0. A total of 223 molecular descriptors—including molecular properties, functional group counts, charge, and topological and geometrical descriptors—were calculated. The resulting sparse model contained only five molecular descriptors and two latent variables. This model successfully predicted whether a material supported or inhibited swarming for each material tested (fig. S6). Modeling biological responses from material properties has previously been achieved (*23*, *31*, *32*). The molecular descriptors were related to hydrophilicity and molecular rigidity, suggesting that both molecular properties influenced the ability of a given polymer to inhibit swarming. These properties have previously been associated with bacterial biofilm formation and have successfully been used to predict antibiofilm materials (*24*).

### Poly(HPhOPA) inhibits *Proteus* swarmer cell differentiation

**Fig. 3.**
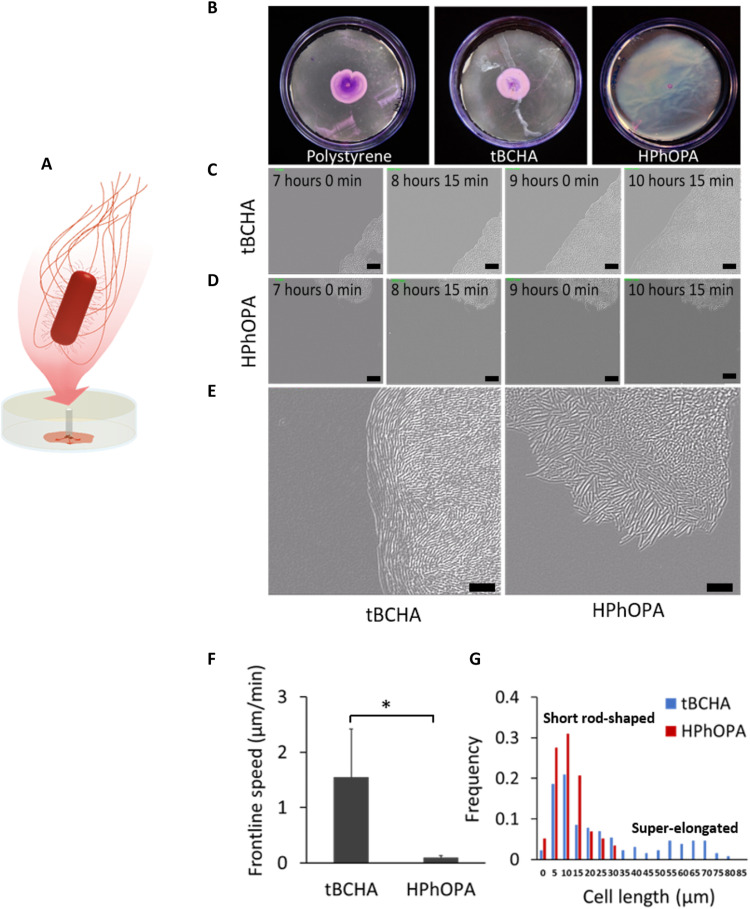
Swarming characteristics of *P. mirabilis* during migration on tBCHA and HPhOPA. (**A**) Schematic depiction of the swarming migration assay. *P. mirabilis* was inoculated onto an uncoated or poly(tBCHA)- or poly(HPhOPA)-coated polystyrene surfaces and incubated at 37°C for 7 hours. (**B**) Images of crystal violet–stained bacteria swarming between agar and, from left to right, uncoated polystyrene, tBCHA-coated, or HPhOPA-coated polystyrene. (**C** and **D**) DIC microscopy time series showing images taken every 45 min from 7 hours after inoculation and for three additional time points on (C) poly(tBCHA) and (D) poly(HPhOPA). Scale bars, 20 μm. (**E**) Enlarged DIC image of the swarming migration front of *P. mirabilis* on poly(tBCHA) (left) or poly(HPhOPA) (right) showing the elongation and alignment of bacterial cells at the moving front on poly(tBCHA) and their absence on HPhOPA. (**F**) Frontline speed and the cell length (**G**) within the cell population found on the poly(tBCHA)-coated (blue) or poly(HPhOPA)-coated (red) surfaces. Error bars in (F) are equal to ± 1 SD for at least three independent replicates. *P ≤ 0.05. Significance was determined by unpaired Student *t* test. Scale bars, 20 μm.

Polystyrene surfaces were coated with either poly(HPhOPA) or poly(tBCHA) and overlaid with agar. Figure 3 (A and B) shows that *P. mirabilis* migrated radially outward over polystyrene and poly(tBCHA) but not on poly(HPhOPA). A time series of differential interference contrast (DIC) micrographs was captured from the 7-hour postinoculation time point for poly(tBCHA) and poly(HPhOPA) (Fig. 3, C and D). These data plus the expanded DIC images (Fig. 3E) show that the distinctive organization and orientation of elongated *P. mirabilis* swarmer cells at the migration front on poly(tBCHA) were absent on poly(HPhOPA). On poly(tBCHA), the bacterial swarm front moved with a speed of 1.6 ± 0.8 μm/min (Fig. 3F), whereas no swarming front was observed on poly(HPhOPA) (Fig. 3F). Similarly, bacterial cell length calculations confirmed the presence of elongated cells, indicative of the hyperflagellated swarming phenotype (*33*) on poly(tBCHA) but not poly(HPhOPA) (Fig. 3G).

Because bacterial swarming migration requires a surfactant to lower surface tension (*34*), the lipopeptide surfactin (25 μM) was incorporated into the agar to determine whether this could overcome poly(HPhOPA)-mediated swarming inhibition (fig. S7). *P. mirabilis* migration after 24 hours was similar on polystyrene, poly(tBCHA), and poly(HPhOPA) (fig. S7A and movies S1 and S2). However, although surfactin enabled *P. mirabilis* to migrate over poly(HPhOPA) (fig. S7A), no characteristic elongated swarmer cells were observed in contrast to poly(tBCHA) (fig. S7B). The frontline speed on poly(HPhOPA) containing surfactant was ~14-fold slower than that on poly(tBCHA), with or without surfactin (fig. S7C). Scanning electron microscopy (fig. S7D) confirmed the lack of swarmer cells on poly(HPhOPA) and showed that *P. mirabilis* cells orientated perpendicular to the surface on poly(HPhOPA) in the presence of surfactin, suggesting that the slow surfactin-mediated migration on poly(HPhOPA) is probably sliding motility (*34*).

### A tBCHA:HPhOPA copolymer offering combined biofilm, swarming, and biomineralization resistance to mono- and multispecies bacterial communities

Poly(HPhOPA) supported high levels of biofilm similar to silicone for *P. mirabilis* (Fig. 1C and fig. S1) and also for *Ps. aeruginosa* and *S. aureus*, although lower levels were observed on uropathogenic *E. coli* (UPEC) (*21*, *22*). Consequently, we sought to determine whether a tBCHA-HPhOPA copolymer could resist biofilm formation by multiple pathogens while retaining swarming inhibitory properties. Three copolymers with tBCHA:HPhOPA monomer feed molar ratios of 4:1, 3:2, and 2:3 were produced using thermally induced catalytic charge transfer polymerization (*27*). The isolated purified copolymers were characterized by ^1^H–nuclear magnetic resonance (NMR) and gel permeation chromatography (GPC), which gave polymer composition ratios of 2.4:1, 2.4:3, and 0.9:3, respectively (figs. S8 and S9 and tables S2 and S3). For the 2.4:3 copolymer, TOF-SIMS (fig. S4 and table S1) confirmed that no micron-scale phase separation had occurred and that both monomer groups were present at the copolymer surface. Figure S10 shows the structures of the cross-linked homo- and heteropolymers formed.

**Fig. 4.**
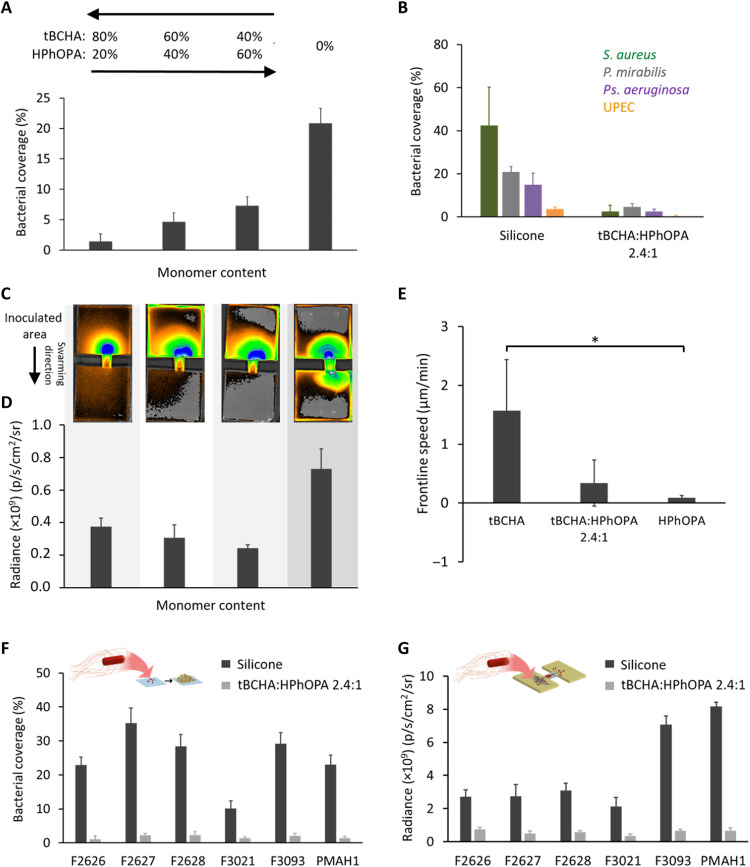
Biofilm and swarming resistance of tBCHA:HPhOPA copolymers. (**A**) *Proteus* biofilm surface coverage on the tBCHA:HPhOPA copolymer series compared with silicone (0%). (**B**) *S. aureus* SH1000 (dark green), *P. mirabilis* (gray), *Ps. aeruginosa* PAO1 (purple), and *E. coli* (orange) surface coverage on silicone compared with tBCHA:HPhOPA 2.4:1. (**C**) Fluorescence images of the *Proteus* catheter bridge swarming assays for the copolymer series and uncoated silicone. (**D**) Quantification of the corresponding fluorescence images for the lower agar block following swarming migration. (**E**) Frontline swarming speed of *Proteus* observed on coatings of tBCHA, HPhOPA, and tBCHA:HPhOPA 2.4:1. Error bars are ± 1 SD unit for at least three independent replicates. *P < 0.05. Significance was determined by one-way ANOVA analysis using Tukey’s multiple comparisons test. (**F**) Schematic depiction of biofilm assay and bacterial surface coverage determined for uncoated silicone or the copolymer with six *dsRed*-labeled *P. mirabilis* clinical isolates. (**G**) Schematic depiction of swarming assay and fluorescence determined at the surface of the lower block following swarming migration of the *P. mirabilis* clinical isolates across either silicone or the tBCHA:HPhOPA 2.4:1 copolymer. For experiments in (F) and (G), SD values are based on the mean value of three biological replicates.

Atomic force microscopy (AFM) indicated that the homopolymer and tBCHA:HPhOPA 2.4:1 copolymer surfaces exhibited similar levels of roughness of 0.3 to 0.4 nm, measured at the bacterial scale (0.5 μm) (fig. S11). Polymer coatings on silicone segments exhibited no delamination or embrittlement after 1 month at 80°C. The lowest *P. mirabilis* biofilm surface coverage of 1.4 ± 1.2% was observed on the tBCHA:HPhOPA 2.4:1 copolymer coating (Fig. 4A). This increased to 4.6 and 7.3%, respectively, as the content of HPhOPA was increased to 40 or 60%. All copolymers resulted in a statistically significant (*P* < 0.001) reduction in bacterial surface coverage compared with silicone (20.8 ± 2.5%) (Fig. 4A). On the tBCHA:HPhOPA 2.4:1 copolymer, biofilm surface coverage was <5% for *S. aureus*, *Ps. aeruginosa*, and *E. coli* (Fig. 4B). The copolymer had no effect on bacterial growth (fig. S2B). In the swarming assay, all three copolymers substantially reduced *P. mirabilis* migration compared with silicone (Fig. 4, C and D). Frontline speed was reduced from 1.6 ± 0.8 μm/min on poly(tBCHA) to 0.3 ± 0.4 μm/min for the copolymer (Fig. 4E). The latter also inhibited biofilm formation and swarming for six *Proteus* clinical isolates (Fig. 4, F and G).

Because CAUTI-associated uropathogens, such as *Proteus*, hydrolyze urea resulting in crystalline deposits on catheters (*29*, *35*), biofilm formation and biomineralization on glass were compared with that on poly(tBCHA-HPhOPA) when incubated with *gfp*-tagged *P. mirabilis* in AU and stained with calcein for Ca or Mg minerals. The results are shown in fig. S12 (A to D) where, in contrast to glass, very little biofilm was observed on the copolymer. Figure S12E shows that the large mineral crystal deposits that formed on the glass were absent from the copolymer, although some diffuse crystalline deposits were apparent (fig. S12E). Quantification of mineral deposition indicated that there was ~3-fold less biomineralization on the copolymer (fig. S12F), although the pH of the bulk liquid cultures was similar on both surfaces after incubation with *P. mirabilis* in AU.

**Fig. 5.**
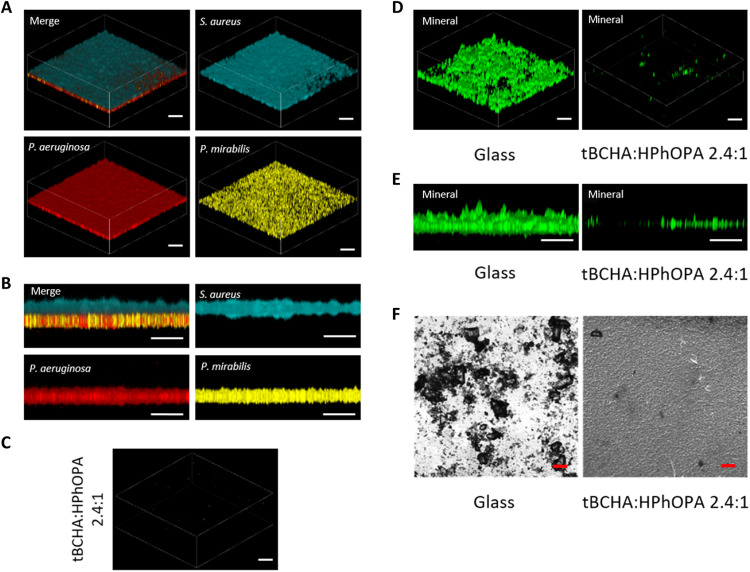
Resistance of the tBCHA:HPhOPA 2.4:1 copolymer to mixed-species biofilm formation and biomineralization in AU. A polymicrobial biofilm was allowed to develop on glass or on the copolymer where colonization over the first 24 hours was with *gfp*-tagged *Proteus*, followed by *mCherry*-tagged *Ps. aeruginosa* PAO1 (red) and *cfp*-tagged *S. aureus* SH1000 (blue) in a 1:1 ratio. After a further 48-hour incubation, the samples were observed via confocal microscopy. (**A**) Three-dimensional (3D) representation of the mixed-species biofilm on glass. (**B**) Transverse view of the mixed-species biofilm. (**C**) 3D representation showing (C) the lack of a mature biofilm on the copolymer. (**D**) 3D representation and (**E**) transverse view of biomineralization on glass and copolymer. (**F**) Bright-field images of biomineralization on glass and copolymer. Scale bars, 50 μm.

Because long-term urinary catheterization generally leads to polymicrobial CAUTIs (*8*), we compared biofilm formation and biomineralization on glass and tBCHA:HPhOPA 2.4:1 copolymer surfaces exposed to a combination of *P. mirabilis*, *Ps. aeruginosa*, and *S. aureus* in AU over 24 hours. Figure 5 (A and B) shows that, on glass, *P. mirabilis* and *Ps. aeruginosa* form an integrated lower biofilm layer covered by a “canopy” biofilm layer of *S. aureus*. This structured biofilm did not form on the tBCHA:HPhOPA 2.4:1 copolymer (Fig. 5C). Furthermore, compared with glass, there was very little biomineralization on the copolymer (Fig. 5, D to F).

### Resistance of poly(tBCHA:HPhOPA) to biofilm formation after preconditioning with urine from catheterized patients

**Fig. 6.**
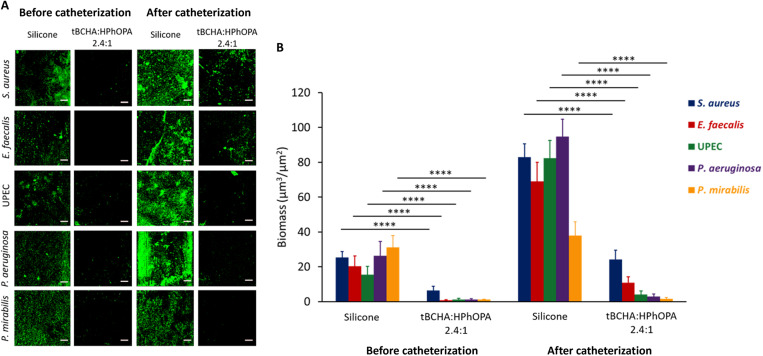
Biofilm formation on silicone and poly(tBCHA:HPhOPA) catheter segments by *S. aureus*, *E. faecalis*, *E. coli* (UPEC), *Ps. aeruginosa*, or *P. mirabilis* after preconditioning with pre- or postcatheterization patient urine (patient 3). (**A**) Representative confocal fluorescence images of *gfp*-tagged bacteria on silicone- or copolymer-coated catheter segments conditioned with pre- and postcatheterization urine. Scale bars, 50 μm. (**B**) Quantification of biofilm biomass for each pathogen. Error bars are equal to ± 1 SD for at least five independent replicates.****P < 0.0001. Significance was determined by two-way ANOVA analysis using Sidak’s multiple comparisons test.

Urinary tract catheterization induces an inflammatory response releasing host proteins into the urine that are deposited onto silicone catheter surfaces, thus enhancing biofilm formation (*18*, *19*). We therefore investigated whether the tBCHA:HPhOPA 2.4:1 copolymer retained its bacterial inhibitory properties after conditioning with urine collected from eight patients undergoing elective colorectal surgery, before and after insertion of a silicone catheter. Indwelling times ranged from 1 to 6 days (table S4). First, biofilm formation by *S. aureus* on silicone catheter segments preconditioned with filtered or unfiltered human urine was compared to ensure that filtration did not alter biofilm formation. No differences were observed (fig. S13). We therefore examined biofilm formation by both Gram-positive (*S. aureus* and *E. faecalis*) and Gram-negative (*Ps. aeruginosa*, *P. mirabilis*, and *E. coli*) bacteria after preconditioning the silicone- and tBCHA:HPhOPA copolymer–coated catheter segments with patient urine. Figure 6A shows a representative series of confocal micrograph images and the corresponding quantitative data for each pathogen (Fig. 6B), demonstrating significantly increased biofilm biomass on silicone conditioned with postcatheterization urine–conditioned surfaces compared with the precatheterization urine for each pathogen except *P. mirabilis*, where only a small increase in biofilm biomass was observed (from patient 3; table S4). A similar trend (increased biofilm formation) was observed for each of the eight patients’ urine samples incubated with *S. aureus*, as shown in fig. S14. It is also clear that compared with silicone, the biofilm inhibitory properties of the tBCHA:HPhOPA 2.4:1 copolymer were retained for each uropathogen tested after conditioning with pre- or postcatheterization urine (Fig. 6).

The increased biofilm biomass observed after surface conditioning with urine, particularly for *S. aureus* and *E. faecalis*, is likely to be related to Fg binding because both pathogens express specific cell wall receptors for this host protein (*18*, *19*). Consequently, we compared Fg deposition on silicone and tBCHA:HPhOPA 2.4:1 copolymer to determine whether differential adsorption was apparent. No cross-reactivity between anti-Fg antibody and bacterial cells in the absence of Fg was observed (fig. S15). Figure S16A shows that when incubated with a range of concentrations of human Fg, the copolymer was much more refractory to Fg adsorption than silicone. Furthermore, the *S. aureus* biofilm biomass formed on silicone conditioned with Fg (0.1 mg/ml) was ~4-fold greater than on tBCHA:HPhOPA 2.4:1 copolymer (fig. S16B). After incubation with postcatheterization filter-sterilized urine from each of the eight patients, a mean of fivefold greater Fg was detected on the silicone surface than on tBCHA:HPhOPA 2.4:1 copolymer (fig. S16C). These data are consistent with Fg deposition being responsible for the significantly increased biofilm biomass for *S. aureus* on silicone and the smaller increases noted for tBCHA:HPhOPA 2.4:1 copolymer.

### Biocompatibility of poly(tBCHA:HPhOPA)

The cytotoxicity of the copolymer was assessed by culturing human fibroblasts with growth media conditioned for 8 days by incubation with coverslips coated with tBCHA:HPhOPA 2.4:1. No differences in metabolic activity or proliferation, as determined using the 3-(4,5-dimethylthiazol-2-yl)-2,5 diphenyltetrazolium bromide (MTT) assay when the mammalian cells were exposed to conditioned or unconditioned media, were observed (fig. S17).

## DISCUSSION

To our knowledge, biocompatible and mechanically flexible materials capable of preventing swarming migration, biofilm formation, encrustation, and host Fg deposition by single or mixed populations of uropathogens have not previously been identified. From a number of potential candidate materials, we selected poly(tBCHA) for further investigation and scale-up because this polymer effectively resisted biofilm formation by *P. mirabilis* and other uropathogens (*21*, *22*), maintained low *F*_PM_ values when polymerized with comonomers that promoted bacterial biofilm formation, was cheap and scalable, and did not promote biomineralization in AU, thus fulfilling two of the criteria desirable for preventing CAUTIs.

Flagella-dependent swarming migration enables *P. mirabilis* to gain entry from a catheter directly to the bladder epithelium and kidneys, potentially causing life-threatening infections (*15*, *36*). It also facilitates the migration of other bacterial species over catheter surfaces (*17*). Despite inhibiting biofilm formation, poly(tBCHA) was unable to prevent *P. mirabilis* swarming. Consequently, we evaluated additional acrylates with diverse pendant groups for swarming inhibition. While the homopolymer of poly(HPhOPA) inhibited swarming, *P. mirabilis* readily migrated over polymerized HPhOPA analogs that lacked either the hydrophilic hydroxyl moiety [poly(PhMA)] or the hydrophobic phenyl group [poly(GDGDA)]. However, poly(HPhOPA) analogs in which the hydroxyl was replaced with an equivalent hydrophilic moiety such as ethylene glycol [as in poly(PEGPHEA)] or where the hydrophobic benzyl group was replaced with a fluorocarbon chain [as in poly(TDFHNA)] preserved the antiswarming properties of the polymer. From this limited monomer series, we derived a PLS categorical regression model from a set of molecular descriptors that predicted whether a specific polymer could inhibit swarming. This model suggested that the interplay between molecular rigidity and hydrophilicity influenced the ability of a polymer to prevent *P. mirabilis* swarming. Previously, we used a similar approach to model *Ps. aeruginosa* biofilm formation on polyacrylates with hydrocarbon pendant groups. For such materials, biofilm formation correlated with a parameter (α) derived from the number of rotatable bonds and the calculated monomer logP value (*23*, *24*). On this class of weak amphiphilic polyacrylates with cyclic and hydrophobic pendant groups that include poly(tBCHA), *Ps. aeruginosa* and other pathogens were unable to form biofilms. Poly(HPhOPA) is, however, chemically distinct from the polymers previously shown to inhibit biofilm formation by the presence of a pendant hydroxyl. This is reflected in the PLS model for swarming inhibition where the hydrophilicity component was more complicated than for the α parameter, and an interplay of both hydrophilic (nOH) and hydrophobic groups (nR═Ct and nCP; fig. S6) was required to successfully predict whether a given polymer material was likely to inhibit swarming.

On poly(HPhOPA), the distinctive organization and orientation of elongated *P. mirabilis* swarmer cells at the migration front observed on poly(tBCHA) were absent (*15*). Swarming motility depends on both flagella and a surfactant to reduce surface tension and promote this distinctive type of bacterial community migration. *P. mirabilis* swarming, but not swarmer cell differentiation, depends in part on colony migration factor, a polysaccharide that also contributes to uropathogenicity, although a specific surfactant has not been identified (*37*). On poly(HPhOPA), the exogenous addition of a heterologous surfactant (surfactin) (*34*) facilitated the slow migration of *P. mirabilis*. However, the migration promoted by surfactin was not swarming as no elongated swarmer cells were apparent at the moving colony front and migration appeared due to sliding motility, a phenomenon that allows a bacterial colony to spread away from the inoculation site through colony expansion facilitated by surfactant-mediated reduction of surface tension (*34*). The altered phenotype of *P. mirabilis* on the surfactin-coated polymer suggests that swarming inhibitory properties of poly(HPhOPA) may involve disrupting surfactant production; however, the precise mechanism remains to be established. Although several different signal transduction pathways are known to regulate *Proteus* swarming, precisely how the surface contact signal is transduced remains unknown but is likely to involve either the flagellum or a cell envelope sensory protein such as RcsF (*28*).

Because poly(tBCHA) inhibited biofilm formation but not swarming, we investigated whether copolymerization with HPhOPA could generate a polymer that inhibited the latter without compromising the former. A tBCHA:HPhOPA 2.4:1 copolymer offered the lowest biofilm surface coverage (1.4 ± 1.2%) and a fivefold reduction in frontline speed for *P. mirabilis* compared with silicone, indicating that the two properties were retained in the copolymer. A similar performance was noted for six different clinical *P. mirabilis* isolates. Furthermore, tBCHA:HPhOPA 2.4:1 copolymer effectively prevented biofilm formation by *Ps. aeruginosa*, *S. aureus*, and *E. coli* inoculated as single species. The content of HPhOPA linearly correlated with bacterial biofilm coverage (*R*^2^ = 0.89) for the copolymer series, although no statistical significance was observed between the homopolymer of tBCHA and the tBCHA:HPhOPA 2.4:1 copolymer (*P* = 0.9), suggesting that a minimum threshold of HPhOPA was required to increase biofilm coverage. A weaker linear correlation (*R*^2^ = 0.69) was observed between HPhOPA content and frontline swarming speed, as the relatively small amount of HPhOPA present in the tBCHA:HPhOPA 2.4:1 copolymer produced a disproportionality large decrease in swarming speed. The apparent higher potency of HPhOPA toward decreasing swarming speed over promoting biofilm formation made it the ideal comonomer selection for the hit polymer formulation.

Compared with borosilicate glass, large crystal struvites did not form on the copolymer surface when exposed to *P. mirabilis* in AU. Although there was little biofilm on the copolymer surface compared with glass, the planktonic bacteria grew similarly in the AU bulk phase, reaching an optical density at 600 nm (OD_600_) of 0.34 ± 0.01 and 0.35 ± 0.01, respectively, over 24 hours (fig. S2). These results suggest that, without a robust biofilm on the surface, there was insufficient urease to alkalinize the AU for substantial biomineralization to occur. The enhanced growth of *P. mirabilis* in biofilms has been directly related to biomineralization rather than the overall nutritional environment provided by AU medium (*38*).

Long-term catheterization frequently results in the development of mixed-species catheter-associated biofilms (*15*, *36*). However, *P. mirabilis* is not usually the earliest urinary catheter–colonizing microorganism but becomes more abundant over time (*36*). In this context, other uropathogens can enhance *Proteus* urease activity (*5*), and the urease-mediated release of ammonia may nutritionally benefit urease-negative bacteria such as UPEC, while biomineralization could offer protection to other species within multispecies biofilms (*36*). In a *P. mirabilis/Ps. aeruginosa* dual-species biofilm formed in AU, the *Proteus* biomass correlated with the localization of mineral deposits and dominated the biofilm. In the absence of biomineralization, *Ps. aeruginosa* dominated, suggesting that ureolytic biomineralization was responsible for the increased success of *P. mirabilis* in mixed-species biofilms (*38*).

Urinary tract catheterization induces a specific inflammatory response resulting in the release of host proteins such as Fg that accumulate in the bladder and deposit on an inserted urethral catheter (*18*, *19*). This, in turn, potentiates infection and enables Fg-binding pathogens such as *S. aureus* and *E. faecalis* to persist in the urinary tract despite a vigorous host response (*18*, *19*). Here, we report that biofilm formation by *S. aureus*, *E. faecalis*, *Ps. aeruginosa*, and *E. coli*, but not *P. mirabilis*, was greatly enhanced on silicone to a far greater extent after conditioning with patient urine after catheterization compared with before catheterization. This contrasts with the tBCHA:HPhOPA 2.4:1 copolymer that retained its biofilm prevention properties. Our data also correlate with the reduced adsorption of Fg on the copolymer compared with silicone and confirm previous studies with respect to greater Fg deposition and *S. aureus* biofilm formation on silicone following catheterization (*18*, *19*). Although some colocalization of *S. aureus* and Fg was apparent in our studies, the biofilm biomass appeared to be more widely distributed, suggesting that other host urine components were likely to be contributing to surface colonization. Further work will be required to identify the host components involved especially for bacteria such as *Ps. aeruginosa* that lack high-affinity Fg receptors and for a greater number of patients and including clinical studies using copolymer-coated catheters.

The resistance to biofilm formation, swarming, biomineralization, and host Fg deposition offered by the tBCHA:HPhOPA 2.4:1 copolymer overcomes several major challenges associated with CAUTIs. Given that the copolymer was not toxic for human fibroblasts, it offers a potential solution for the prevention of such infections but will require further in vivo evaluation to confirm our in vitro and ex vivo clinical findings.

## MATERIALS AND METHODS

### Bacterial strains and growth conditions

*P. mirabilis* strain Hauser 1885, *Ps. aeruginosa* PAO1 (Washington subline, Nottingham collection), *S. aureus* SH1000, *E. faecalis*, and UPEC strain 536 were included. *Proteus* isolates F3021, F2626, F2627, F2628, and F3093 were isolated at Queen’s Medical Centre, Nottingham, UK, from patients with urinary tract infections. Bacteria were routinely grown at 37°C in LB or RPMI 1640 (Sigma-Aldrich) with shaking at 200 rpm or on LB agar (2%, w/v). For biofilm experiments, bacteria were also cultured in AU (*39*). Where required, plasmids for constitutively expressing fluorescent proteins green fluorescent protein (GFP) (pBK-miniTn7-*egfp*) (*40*), DsRed (pBK-miniTn7-*dsRed*) (*40*), cyan fluorescent protein (CFP) (pTKP004-CPF) (*41*), and mCherry (pMMR) (*42*) were introduced into the relevant host strain by conjugation or electroporation.

### Polymer microarrays

All chemicals were used as received from Sigma-Aldrich, Polysciences, or Alfa Aesar. Monomers DHPA and DMDA were prepared as previously described (*43*). ^1^H-NMR of the monomers is shown in fig. S9. Polymer microarrays were prepared as before (*22*) using an XYZ3200 dispensing workstation (Biodot) to print monomers onto poly(2-hydroxyethyl methacrylate)–coated glass slides (Genetix) before irradiation with long-wave UV light. Monomer solutions consisted of 75% (v/v) monomer in dimethyl formamide with 1% (w/v) 2,2-dimethoxy-2-phenylacetophenone as photoinitiator. Polymer acronyms are as follows: HDFDA, BAEDA, tBCHA, ethylhexyl acrylate (EHA), octafluoropentyl methacrylate (OFPMA), ethylene glycol phenyl ether methacrylate (EGPEMA), ethylene glycol phenyl ether acrylate (EGPEA), 2-hydroxy-3-phenoxypropyl acrylate (HPHOPA), *bis*[2-(methacryloyloxy)ethyl] phosphate (BMAOEP), TMSiA, poly(ethylene glycol) phenyl ether acrylate (PEGPHEA), trimethylolpropane triacrylate (TMPTA), hexanediylbis[oxy(2-hydroxy-3,1-propanediyl)] bisacrylate (HBOPBA), chloro-2-hydroxy-propyl methacrylate (ClHMPA), CMAOE, dimethylamino-propyl acrylate (DMAPA), tetra(ethylene glycol) diacrylate (TEGDA), di(ethylene glycol) ethyl ether acrylate (DEGEEA), hydroxybutyl acrylate (HBA), poly(propylene glycol) acrylate (PPGA), hydroxypropyl acrylate (HPA), and hydroxyethyl acrylate (HEA).

### Coverslip, catheter, and petri dish polymer coating

Coverslips and petri dish polymer coatings were prepared as before (*22*). Polymer-coated catheter segments for bacterial biofilm and swarming assays were prepared from longitudinally cut 1.5-cm-length sections of Foley silicone urinary catheters (size Fr. 22, Bard Medical). Catheter segments were treated in a custom-built plasma barrel chamber with oxygen plasma for 5 min at 50 W and pO_2_ of 300 mbar and immersed in monomer solutions for 10 s before photopolymerization for 1 min, with O_2_ < 2000 parts per million. Presynthesized polymer was directly dip-coated onto silicone catheter segments as a 5 weight % solution in toluene:chloroform. The samples were dried for a further 1 hour before being placed under vacuum (0.3 mbar) for 24 hours.

### Synthesis and analysis of tBCHA:HPhOPA copolymers

To generate tBCHA:HPhOPA copolymers with different monomer ratios, the appropriate volumes of each monomer required to deliver the target reagent feed molar ratio were placed in a dried flask such that the total volume of the monomer was 12 ml. Azobisisobutyronitrile (28.8 mg), bis[(difluoroboryl)diphenylglyoximato] cobalt (II) (12 mg), and 24 ml of toluene were then added with stirring, and the mixture was degassed for 30 min with dry nitrogen. The flask was heated to 70°C, and polymerization was allowed to proceed for 24 hours. The flask was cooled to prevent further reaction, and the polymers were purified by precipitation twice in methanol and twice into petroleum ether before drying under vacuum. Polymer chemical composition was determined by ^1^H-NMR on a Bruker DPX400 Ultra-Shield spectrometer. Chemical shifts were reported in parts per million (δ units) downfield from internal tetramethylsilane or deuterated chloroform, and the spectra were analyzed using MestReNova 6.0.2 (Mestrelab Research S. L.). GPC was performed to determine polymer molecular weight and polydispersity on a Polymer Labs GPC50 Plus fitted with a differential refractometer (RO), capillary viscometer (DP), and dual-angle laser light-scattering (15 and 90) detectors on a Resipore Mixed-D column. Polymer chemical compositions are reported in tables S2 and S3 and fig. S8. To assess the strength of the interface between the polymer coating and silicone catheter segments, coated samples were subjected to a rolling tube compression test as previously described (*44*).

### Atomic force microscopy

Coated surfaces were analyzed using a Bruker Dimension Icon AFM (BruckerNano) using PeakForce QNM mode with Bruker MSNL-F tips. Three different areas were scanned for each sample with the field of view ranging from 50 nm to 20 μm. Root mean square values for roughness were calculated using an average of at least five measurements over areas of 0.5 μm × 0.5 μm.

### Time-of-flight secondary ion mass spectrometry

TOF-SIMS measurements were conducted using a TOF-SIMS IV (IONTOF GmbH) instrument operated using a 25-kV Bi_3_^2+^ primary ion source exhibiting a pulsed target current of >0.3 pA. Samples were scanned at a pixel density of 100 pixels/mm, with eight shots per pixel over a given area. An ion dose of 2.45 × 10^11^ ions/cm^2^ was applied to each sample area ensuring static conditions were maintained throughout. Both positive and negative secondary ion spectra were collected (mass resolution of >7000 at a mass/charge ratio of 29), over an acquisition period of 15 scans. The nonconductive nature of the samples required that charge compensation was applied via a low-energy (20-eV) electron floodgun. Peaks were identified using a peak search tool (SurfaceLab 6), minimum counts were set to 100, and maximum background was set to 0.8.

### Bacterial biofilm formation

Bacterial biofilms were grown on polymer array slides as described before (*21*, *22*). Briefly, UV-sterilized polymer slides were placed in petri dishes containing 15 ml of AU medium (*39*) and inoculated with diluted (OD_600_ = 0.01) *dsRed*-tagged *P. mirabilis* from overnight cultures grown at 37°C and 60 rpm in AU. As growth medium controls, the slides were also incubated without bacteria. Following a 72-hour incubation, slides were washed three times with 15 ml of phosphate-buffered saline at room temperature for 5 min. After rinsing with distilled H_2_O to remove salts and being air-dried, the fluorescent images from the slides incubated in sterile media or in media containing bacteria were acquired using a GenePix Autoloader 4200AL Scanner (Molecular Devices) with a 555-nm excitation laser. The total fluorescence intensity from polymer spots was acquired with GenePix Pro 6 software (Molecular Devices) using Eq. A where *F*_test_ is the fluorescence intensity measured per unit area after incubation with bacteria and *F*_control_ is the fluorescence intensity measured per unit area measured on arrays incubated in growth medium alone

$$F=F_{\mathrm{test}}-F_{\mathrm{control}}$$ (A)

Polymer-coated coverslips or catheter segments were incubated with fluorescently tagged bacteria (*gfp*-tagged *P. mirabilis*, *mcherry*-tagged *Ps. aeruginosa*, and *cfp*-tagged *S. aureus*) in mono- or mixed-species cultures at 37°C with shaking at 60 rpm for 72 hours in either RPMI, RPMI following conditioning of catheter segments or polymer-coated coverslips by incubating in AU for 72 hours, or in AU. Air-dried samples were examined using a Carl Zeiss LSM 700 laser scanning confocal microscope fitted with 405-, 488-, and 555-nm excitation lasers and a 10×/numerical aperture (NA) 0.3 objective. Images were acquired using ZEN 2009 imaging software (Carl Zeiss). Bacterial surface coverage was quantified using ImageJ 1.44 software [National Institutes of Health (NIH), USA]. For mixed-species biofilm formation, polymer-coated coupons were incubated with AU (15 ml) containing 2 μM calcein (Sigma-Aldrich) and inoculated with *P. mirabilis*, *Ps. aeruginosa*, and *S. aureus* mixed in a 10:1:1 ratio. Biomass and mineralization were quantified using ImageJ (NIH, Bethesda, MD, USA) and Comstat 2.1 (www.comstat.dk).

### Swarming motility

Catheter bridge swarming assays were modified from (*17*). Two perpendicular 1.5-cm channels were cut out of 1% LB agar and bridged by catheter segments (fig. S3). To assess the migration of *P. mirabilis* over silicone-coated catheter sections, aliquots (10 μl) of cultures grown in LB for 4 hours at 37°C were inoculated adjacent to the edge of central channels cut in the LB agar. Inocula were dried for 10 min before catheter sections were placed across the channel adjacent to the *P. mirabilis* inocula.

After a 16-hour incubation at 37°C, the migration of fluorescently labeled bacteria across the catheter bridges was quantified using an IVIS Spectrum (PerkinElmer). *Proteus* swarming assays were also performed at the interface between LB agar and the surface of a polystyrene petri dish with or without coating with HPhOPA or tBCHA. Where required, 25 μM surfactin (Sigma-Aldrich) was incorporated into the agar. Following incubation, the agar was removed, and swarm colonies were stained with 0.05% (w/v) crystal violet. Swarming was also imaged over time at the interface between uncoated or polymer-coated coverslips and LB agar by bright-field DIC microscopy (60×, NA = 1.4) using an inverted microscope (TE2000, Nikon, Japan) equipped with an OKOLab (Italy) incubation system. The frontline speed of bacterial swarming migration was calculated using Eq. B

$${\vec{v}}_{i}=|\vec{x}(t_{i+1})-\vec{x}(t_{i})|/\Delta t$$

where ∆*t* = *t*_*i* + 1_ − *t_i_*, while the bacterial average speed is calculated using Eq. C

$${\vec{v}}_{\mathrm{average}}=\frac{1}{n}\sum_{i=1}^{n}({\vec{v}}_{i})$$ (B)

where *n* is number of points in the track. *P. mirabilis* swarmer cell differentiation was also visualized by scanning electron microscopy after fixation using FEI Tecnai G2 12 Biotwin and JEOL 7100F FEG-SEM electron microscopes, respectively.

### PLS regression

To assess the relationship between the molecular structures of the monomers and their swarming inhibitory properties, PLS was performed using calculated monomer molecular descriptors. Polymers that inhibited swarming were assigned a value of 1, and noninhibitory polymers were assigned a value of 0. A total of 223 molecular descriptors—including molecular properties, functional group counts, constitutional, charge, and topological and geometrical descriptors—were calculated from the Simplified Molecular Input Line Entry System strings to represent the monomer structures using the Dragon 7.01 package. PLS regression was performed using a bespoke MATLAB script. Initially, four latent variables were used as determined from the root mean square of cross validation (RMSECV) curve using the leave-one-out method. Four latent variables were identified from the RMSECV curves for PLS models constructed with both 223 X variables and for a sparse model with 9 X variables (fig. S6). The total number of descriptors was sequentially reduced by taking the top 10% of descriptors with the greatest absolute regression coefficient to decrease the number of X variables to a similar number of the Y variables, avoiding overfitting (*32*). This was repeated until the PLS model no longer successfully predicted swarming. The final model contained five molecular descriptors and two latent variables. The reduced number of latent variables was used to ensure no overfitting.

### Collection of human urine

To determine the impact of surface conditioning by urine on biofilm formation, pre- and postcatheterization urine samples were obtained from eight patients (table S4) undergoing elective colorectal surgery at the Queen’s Medical Centre, Nottingham University Hospitals NHS Trust, UK (NUHNT) who had consented to the study. The study was approved by the NUHNT Research Ethics Committee, under the Integrated Research Application System project ID: 232293.

### Bacterial biofilm formation on Fg- and urine-conditioned catheter sections

Silicone- and polymer-coated catheter segments were incubated for 16 hours at 4°C with human Fg (0.01, 0.1, or 1 mg/ml; Sigma-Aldrich) or human urine with or without filter-sterilizing via a 0.22-μm membrane filter (Millipore). The Fg- and urine-conditioned catheter segments were incubated with *gfp*-tagged *S. aureus* at 37°C for 24 hours in RPMI, and the background was blocked with 10% human serum, followed by a rabbit antibody to Fg (Invitrogen) and then goat anti-rabbit QDot700 conjugate (Invitrogen). Both primary and secondary antibodies were tested to ensure that there was no cross-reactivity with *S. aureus* (fig. S14). Samples were examined by confocal microscopy (Carl Zeiss LSM 710). Surface coverage, biomass, and Fg deposition were determined using ImageJ (NIH, Bethesda, MD, USA) and Comstat 2.1. (www.comstat.dk).

### MTT viability assay

The MTT cell viability assay was adapted from Mossmann (*45*). MRC-5 cell lines (American Type Culture Collection) were cultured in minimum essential medium (MEM) supplemented with 1% nonessential amino acids, Hepes, 1% l-glutamine, 1% penicillin, 1% streptomycin, and 10% fetal bovine serum. Conditioned medium was generated by incubation of poly(tBCHA:HPhOPA)-coated coverslips in supplemented MEM for 8 days to allow soluble components to leach from the polymer. MRC-5 cells were incubated in 96-well plates with fresh or conditioned media at a range of dilutions. MTT assays were carried out for 72 hours at 37°C in 5% CO_2_ by adding 50 μl of MTT solution (2 mg/ml) to each well. Cells were incubated for a further 3 to 4 hours to allow formazan production. The aqueous medium was aspirated, the formazan product was solubilized in dimethyl sulfoxide (150 μl per well), and absorbance was measured at *A*_550_ (absorbance at 550 nm) and normalized to cell density.

### Statistical analysis

Results are presented as means ± SEM. Statistical calculations and tests were performed using Student’s *t* test and one- or two-way analysis of variance (ANOVA) to determine the significance of differences where appropriate.

## Supplementary Materials

### This PDF file includes:

Figs. S1 to S17 Tables S1 to S4 References

## Notes

View/request a protocol for this paper from *Bio-protocol*.
