# Supplementary material for: Discovery of a polymer resistant to bacterial biofilm, swarming, and encrustation
Source: Sci Adv. 2023 Jan 25;9(4):eadd7474. doi: 10.1126/sciadv.add7474 (PMC9876547; doi:10.1126/sciadv.add7474)
Supplement: Supplementary file 1 — Figs. S1 to S17 Tables S1 to S4 References [file sciadv.add7474_sm.pdf]

Science Advances
AAAS

Supplementary Materials for

Discovery of a polymer resistant to bacterial biofilm, swarming, and encrustation

Jean-Frédéric Dubern *et al.*

Corresponding author: Paul Williams, paul.williams@nottingham.ac.uk

Sci. Adv. **9**, eadd7474 (2023)
DOI: 10.1126/sciadv.add7474

The PDF file includes:

Figs. S1 to S17
Tables S1 to S4
Legends for movies S1 and S2
References

Other Supplementary Material for this manuscript includes the following:

Movies S1 and S2

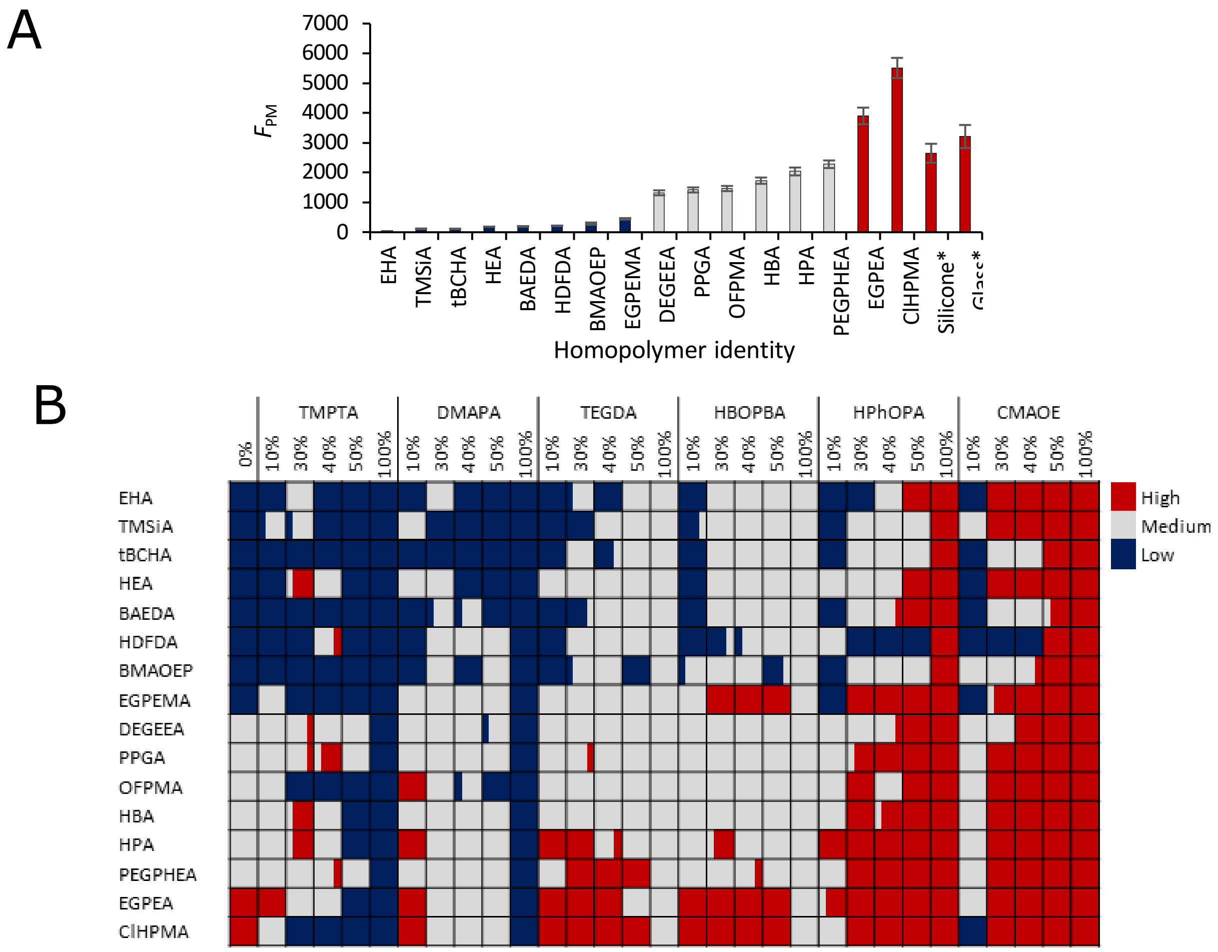

Fig. S1. *Proteus mirabilis* (DsRed labelled) biofilm formation on the polymer array as quantified by fluorescence (F_{PM}). ***(A)*** F_{PM} measured for homopolymers of candidate monomers. Monomer structures are shown in **Fig. 1*B***. Samples have been colored according to categories of high (red, F_{PM} > 2,500), medium (grey, 2,500 > F_{PM} > 500) or low (blue, F_{PM} < 500) bacterial attachment. *Values for silicone and glass are estimates based upon scaled up coverage measurements and comparison with tBCHA. ***(B)*** F_{PM} obtained on the copolymer microarray, represented in categories of bacterial attachment in (***A***) and as indicated on the color scale. For each sample, the centre of the associated square is colored according to the mean value (n=3), whilst the left and right portions are respectively colored ± standard deviation.[1]

[1] The 8 low attachment monomers produced low attachment copolymers when mixed with similarly low attachment monomers (TMPTA and DMAPA). A few monomers acted synergistically such that copolymers of TMPTA with either EHA, HEA or HDFDA exhibited high attachment whilst the homopolymers remained low attachment. Bacterial biofilm formation generally reduced on copolymers of medium and high attachment controls when mixed with low attachment test monomers TMPTA and DMAPA. OFPMA was particularly susceptible to reduced bacterial attachment, with low bacterial attachment observed on the copolymer containing 30 % (v/v) of TMPTA, whilst medium to high attachment was observed on all copolymers with monomers containing long chain glycols (PPGA and PEGPHEA) up to 50% (v/v).
Copolymerisation with the medium attachment test monomers produced similar results across all hit monomers with the exception of EGPEMA, which produced copolymers with medium to high attachment, observed after addition of only 10% (v/v) of the test monomer.
Addition of the hit monomers with the high attachment test monomers enabled discrimination of the ability of these monomers to maintain low bacterial attachment once diluted. Monomers tBCHA, BAEDA and HDFDA all achieved low to medium bacterial attachment when copolymerised with either HPHOPA or CMAOE up to addition of 40% of the test monomer and were thus the monomers of choice for the creation of a multi-functional copolymer with the desired microbiological properties.

A

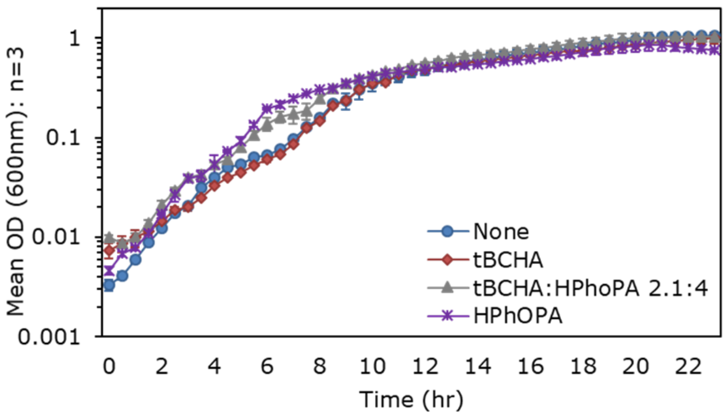

B

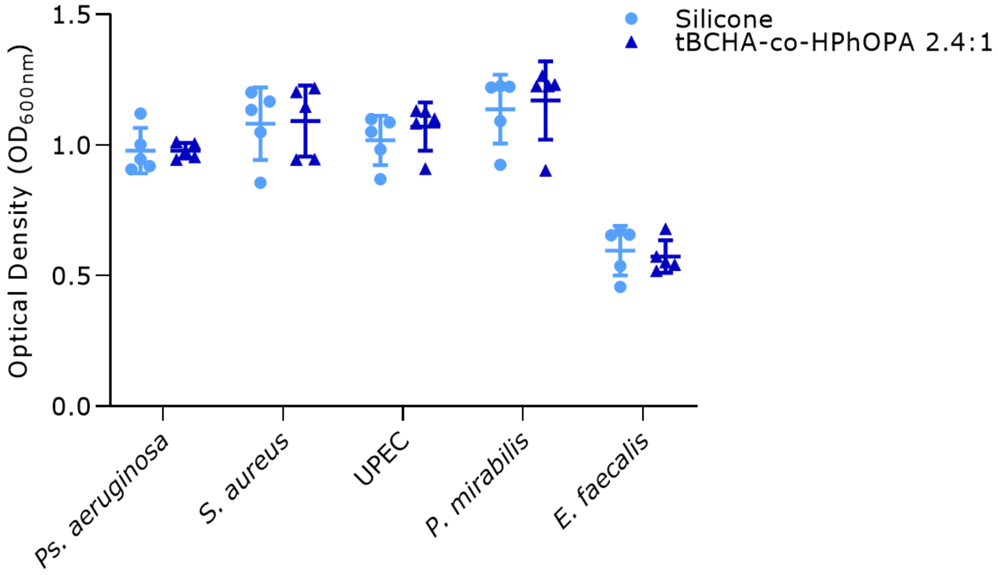

Fig. S2. (**A**) The homopolymers tBCHA, HPhOPA and copolymer tBCHA:HPHOPA 2.4:1 do not inhibit *P. mirabilis* growth. *P. mirabilis* 1885 was cultured in the uncoated or coated wells of a 96 well microtitre plate in RPMI-1640 medium and growth monitored by measurement of OD_{600}. Standard deviations are based on the mean values of three parallel cultures. (**B**) The tBCHA:HPhOPA 2.4:1 copolymer does not inhibit bacterial growth. Final cell population densities ($OD_{600\ nm}$) reached by *Ps. aeruginosa*, *S. aureus*, UPEC, *P. mirabilis* and *E. faecalis* cultures exposed to uncoated silicone or tBCHA:HPhOPA 2.4:1 coated silicone catheters for 22 h. Values given are the means of five independent cultures. Error bars are ± SD.

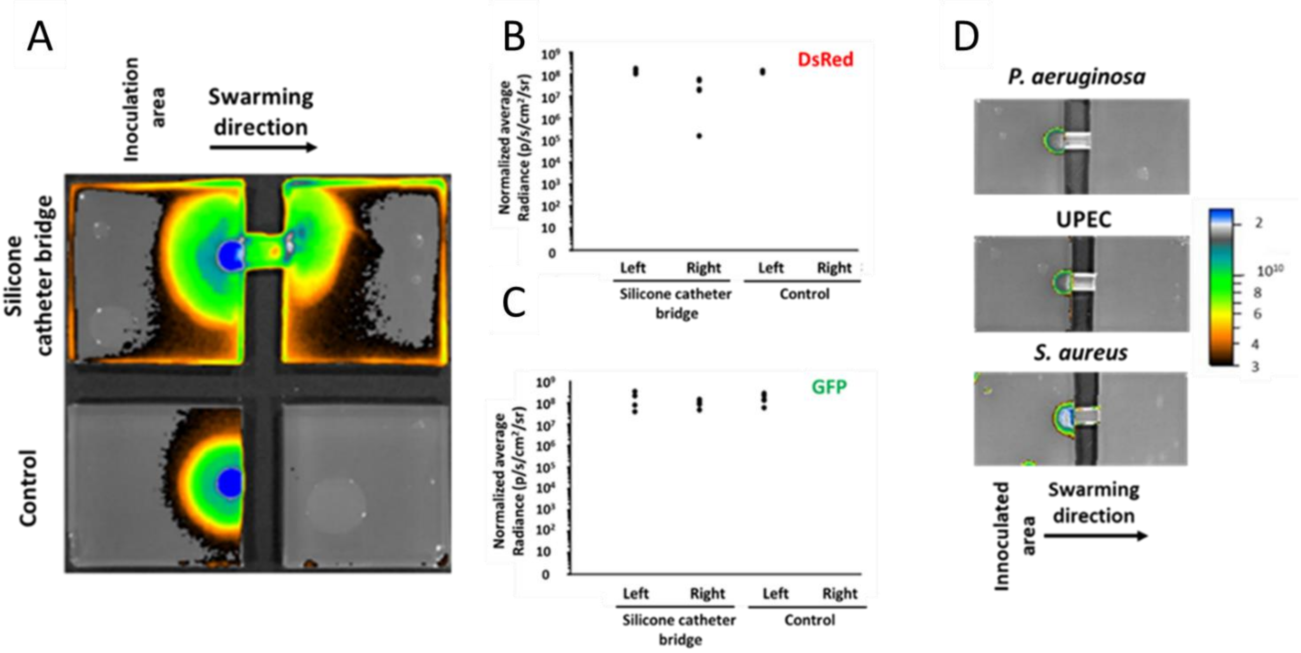

Fig. S3. Catheter bridge swarming migration assay. (***A***) Swarming of *P. mirabilis* 1885 over a silicone catheter bridge. GFP-labelled *P. mirabilis* was inoculated on one side of a catheter bridge linking two unconnected LB agar blocks (upper panel) or lacking a bridge (lower panel) the fluorescence intensity on the lower agar block quantified after incubation for 16 h. Fluorescence radiance quantified on the surface of silicone catheter bridge and control (left and right) agar sections for (***B***) DsRed or (***C***) GFP labelled *P. mirabilis* swarming migration. Results obtained from five independent experiments were normalized to the background autofluorescence of the agar. (***D***) GFP-labelled *Ps. aeruginosa* PAO1, UPEC and *S. aureus* SH1000 are unable to migrate over silicone catheter bridges.

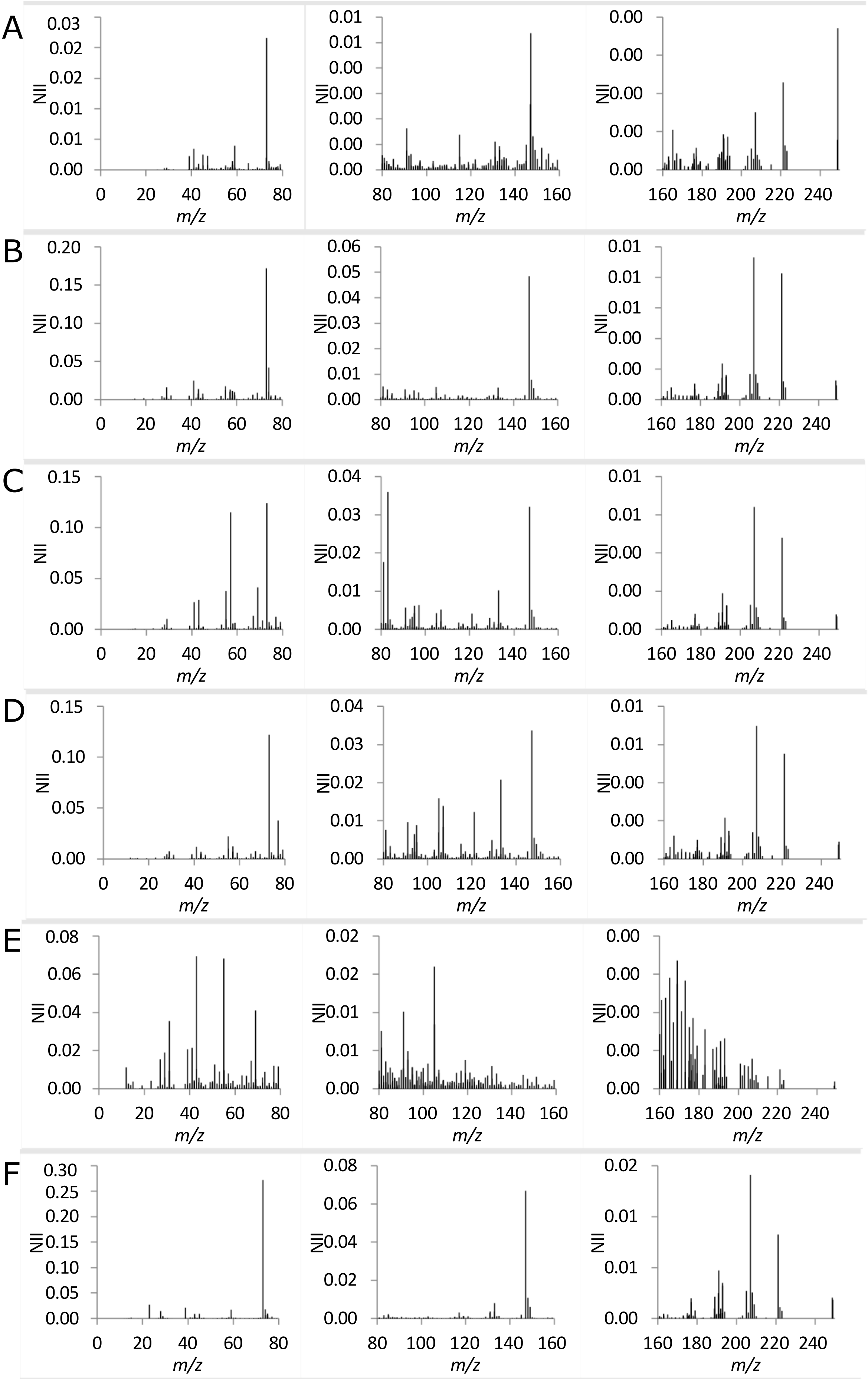
A
B
C
D
E
F
NIII
m/z

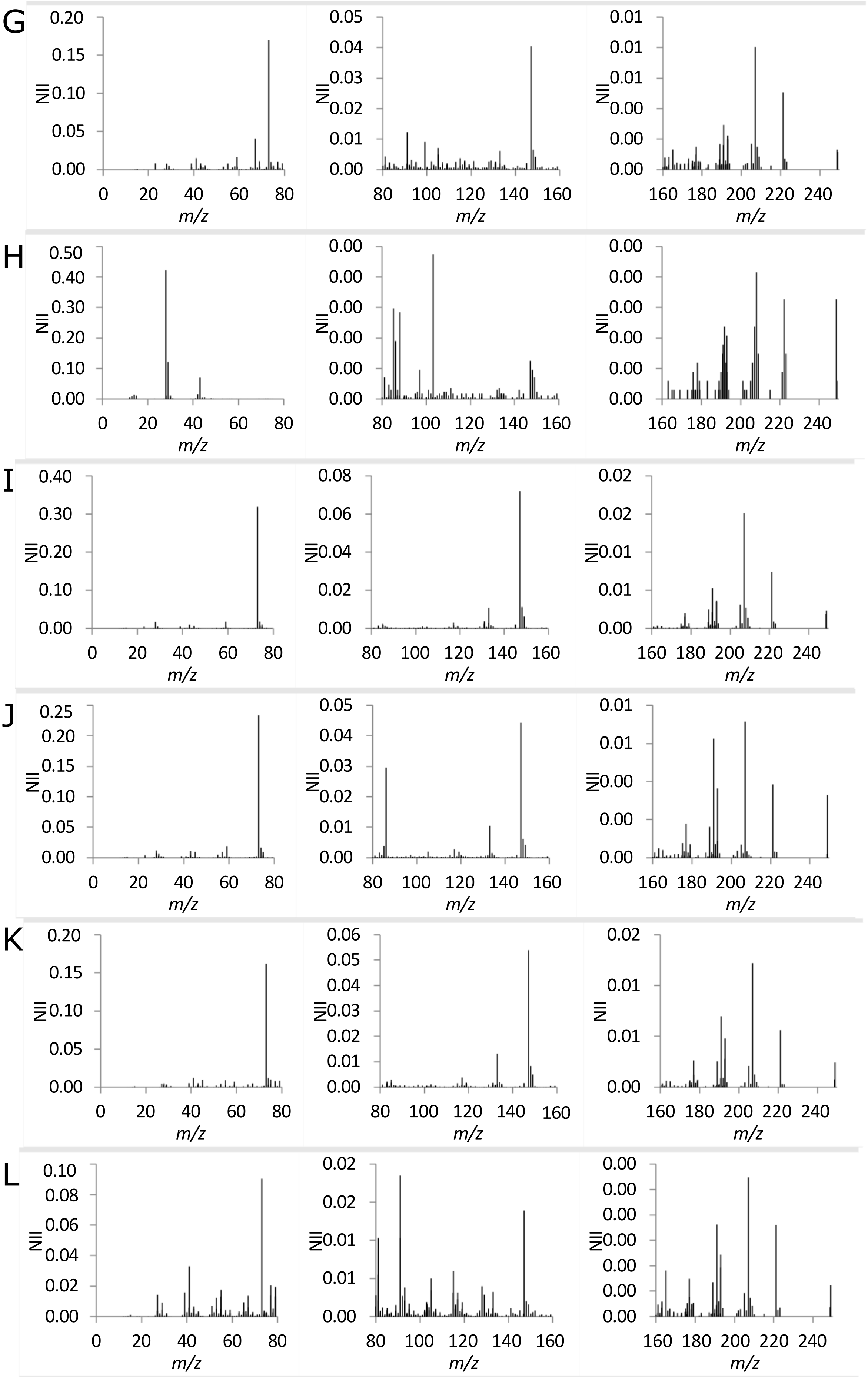
G
H
I
J
K
L
NII
m/z

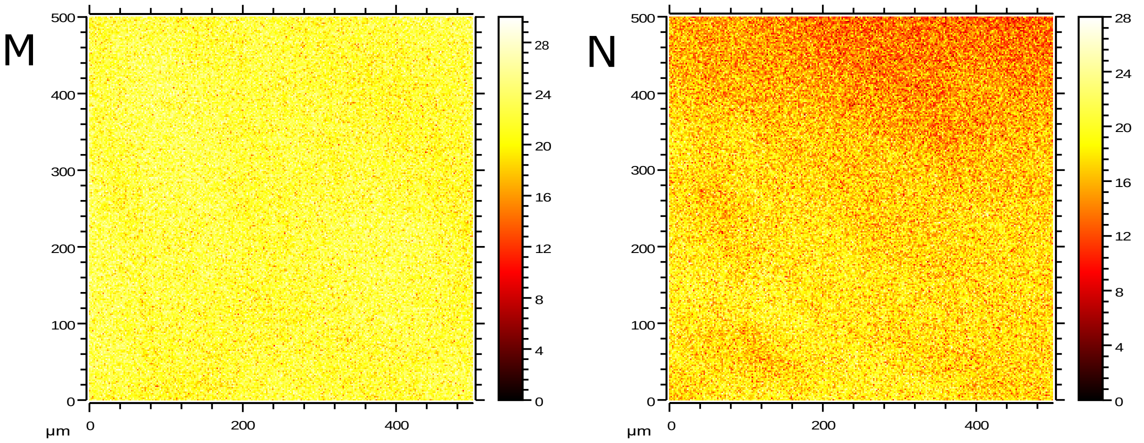

Fig. S4. Positive ToF-SIMS spectra, split into mass regions 0-80, 80-160 and 160-240 m/z with altered normalized ion intensity (NII) ranges for polymers (***A***) BPAPGDA, (***B***) GDGDA, (***C***) tBCHA:HPhOPA 2.4:1, (***D***) HPhOPA, (***E***) TDFHNA, (***F***) pEGPhEA, (***G***) PhMA, (***H***) DHPA, (***I***) PhoPDA, (***J***) DMDA, (***K***) tBCHA, and (***L***) tCdMdA. Text files of the positive and negative spectra for the polymer coatings are included as additional files within the supplementary information. (***M-N***) ToF-SIMS ion images as measured from 500 x 500 µm areas of tBCHA:HPhOPA 2.4:1 for the ions (***M***) $C_4H_9^+$, likely derived from the tert-butyl group on tBCHA, and (***N***) $C_6H_5O^-$, likely derived from the benzyl group on HPhOPA. The ion intensity (counts) is indicated by the intensity scales provided. No micron-scale phase separation of the two monomers was observed on the polymer surface.

ToF-SIMS analysis of polymer coatings

Many polymers exhibited peaks at 74.98 m/z, 147.09 m/z, 207.05 m/z and 221.18 m/z in the positive spectrum, all associated with the silicone, polydimethylsiloxane (PDMS). Thus, the top surface of most of the polymers was contaminated with a thin layer of PDMS oligomers. Different biological behaviours were observed on materials that both contain this contaminant suggesting that either the contaminant is not sufficient to overwhelm the underlying coating chemistry or that the oligomers were washed away in the growth medium during incubation and thus did not impact on the biological performance. Chlorine contamination was also observed on some samples, notably GDGDA, BPAPGDA, pPEGPhEA, PhMA, and tBCHA (**Table S3**). This group of polymers includes materials that both supported and resisted swarming, suggesting that the biological performance of a material had not been compromised.

The high intensity ions observed for each material (**Table S3**) were consistent with the chemical structures of the materials synthesised. For example, ions associated with benzyl rings, including $C_6H_5O^-$, $C_6H_5^+$, $C_7H_5^-$ and $C_6H_{11}^+$, were observed for materials BPAPGDA, HPhOPA, PhMA, pEGPhEA, PhoPDA, DMDA and tCdMdA, all of which contained benzyl rings or cyclic structures. Unsurprisingly, the ions CF^+ and C^- were intense for the polymer of TDFHNA, which contains fluorocarbon moieties.

The tBCHA:HPHoPA 2.4:1 copolymer had a high intensity of ions associated with the acrylate backbone ($C_3H_3O_2^-$ and C_2HO^-), the benzyl group on HPhOPA ($C_6H_5O^-$, $C_6H_5O_4^+$) and the tert-butyl group of tBCHA ($C_4H_9O^+$, $C_4H_9^+$, $C_4H_7^+$) confirming that both monomers were present at the surface of the copolymer coating. The distribution of the ions appeared to be uniform over the polymer surface (Fig. S4*M-N*), suggesting that no micron-scale phase separation of the two monomers had occurred.

Table S1. List of the ions with highest normalised intensity for the different polymer coatings used for swarming assays, with the normalised ion intensity (NII), m/z and likely assignment reported. Note: a number of possible assignments are possible for ions with an m/z > 150. The assignment provided is the chemical structure with the lowest deviation (<75 ppm) that makes chemical sense.

BPAPGDA			GDGDA			tBCHA:HPHoPA (2.4:1)			HPhOPA		
NII	m/z	Assignment	NII	m/z	Assignment	NII	m/z	Assignment	NII	m/z	Assignment
0.0216	73.0657	$C_4H_9O^+$	0.174	34.9723	Cl^-	0.128	93.0408	$C_6H_5O^-$	0.186	93.0408	$C_6H_5O^-$
0.0136	34.9723	Cl^-	0.172	73.0657	$C_4H_9O^+$	0.124	73.0657	$C_4H_9O^+$	0.122	73.0657	$C_4H_9O^+$
0.0101	211.022	$C_9H_7O_6^-$	0.055	36.9692	$^{37}Cl^-$	0.115	57.0678	$C_4H_9^+$	0.0529	71.0133	$C_3H_3O_2^-$
0.0094	74.9837	$CH_3SiO_2^-$	0.0485	147.087	$C_6H_{11}O_4^+$	0.0595	71.0133	$C_3H_3O_2^-$	0.0399	41.0067	C_2HO^-
0.0087	149.005	$C_4H_5O_6^-$	0.0419	74.9837	$CH_3SiO_2^-$	0.0527	41.0067	C_2HO^-	0.0378	77.0348	$C_6H_5^+$
0.0082	89.038	$C_7H_5^-$	0.0284	15.9932	O^-	0.0412	69.0704	$C_5H_9^+$	0.0337	147.087	$C_6H_{11}O_4^+$
0.0081	93.0408	$C_6H_5O^-$	0.0248	41.0403	$C_3H_5^+$	0.0375	55.0534	$C_4H_7^+$	0.0223	55.0148	$C_3H_3O^+$
0.008	41.0067	C_2HO^-	0.0247	71.0133	$C_3H_3O_2^-$	0.036	83.0924	$C_6H_{11}^+$	0.0208	133.057	$C_5H_9O_4^+$
0.0076	135.014	$C_7H_3O_3^-$	0.0234	25.0112	C_2H^-	0.0321	147.087	$C_6H_{11}O_4^+$	0.0206	15.9932	O^-
0.0073	75.0264	$C_6H_3^-$	0.0212	41.0067	C_2HO^-	0.0288	43.0558	$C_3H_7^+$	0.0203	25.0112	C_2H^-
0.0068	25.0112	C_2H^-	0.0175	55.0534	$C_4H_7^+$	0.0266	41.0403	$C_3H_5^+$	0.0159	105.073	$C_4H_9O_3^+$

TDFHNA			pEGPhEA			PhMA			DHPA		
NII	m/z	Assignment	NII	m/z	Assignment	NII	m/z	Assignment	NII	m/z	Assignment
0.237	18.9969	F^-	0.272	73.0657	$C_4H_9O^+$	0.17	73.0657	$C_4H_9O^+$	0.152	15.9932	O^-
0.0694	43.0118	$C_2H_3O^+$	0.0872	15.9932	O^-	0.117	93.0408	$C_6H_5O^-$	0.121	28.9972	CHO^+
0.0682	55.0148	$C_3H_3O^+$	0.0669	147.087	$C_6H_{11}O_4^+$	0.0406	67.0534	$C_5H_7^+$	0.0704	43.0558	$C_3H_7^+$
0.0424	59.0214	$C_2H_3O_2^-$	0.0397	17.0017	HO^-	0.0405	147.087	$C_6H_{11}O_4^+$	0.0599	17.0017	HO^-
0.041	69.0006	$H_2O_3F^+$	0.0283	13.0076	CH^-	0.0294	15.9932	O^-	0.0538	13.0076	CH^-
0.0355	30.9984	CF^+	0.028	25.0112	C_2H^-	0.0287	34.9723	Cl^-	0.0202	25.0112	C_2H^-
0.0279	71.0133	$C_3H_3O_2^-$	0.0271	22.9894	Na^+	0.0224	25.0112	C_2H^-	0.0195	12.0001	C^-
0.0214	41.0403	$C_3H_5^+$	0.0225	34.9723	Cl^-	0.0207	41.0067	C_2HO^-	0.0153	27.9726	Si^-
0.0207	39.0224	$C_3H_3^+$	0.0212	38.963	K^+	0.0173	43.021	$C_2H_3O^-$	0.0142	14.0135	CH_2^+
0.0192	39.0275	$C_3H_3^-$	0.018	74.9837	$CH_3SiO_2^-$	0.015	41.0403	$C_3H_5^+$	0.0114	15.0219	CH_3^+
0.019	28.9972	CHO^+	0.0141	207.049	$C_{10}H_7O_5^+$	0.0143	44.9982	CHO_2^-	0.0113	14.0152	CH_2^-

PhoPDA			DMDA			tBCHA			tCdMdA		
NII	m/z	Assignment	NII	m/z	Assignment	NII	m/z	Assignment	NII	m/z	Assignment
0.319	73.0657	$C_4H_9O^+$	0.234	73.0657	$C_4H_9O^+$	0.277	73.0657	$C_4H_9O^+$	0.0904	73.0657	$C_4H_9O^+$
0.072	147.087	$C_6H_{11}O_4^+$	0.0582	15.9932	O^-	0.0902	15.9932	O^-	0.0586	25.0112	C_2H^-
0.0665	15.9932	O^-	0.0443	147.087	$C_6H_{11}O_4^+$	0.0539	147.087	$C_6H_{11}O_4^+$	0.0344	15.9932	O^-
0.0341	25.0112	C_2H^-	0.0314	25.0112	C_2H^-	0.0437	17.0017	HO^-	0.0329	41.0403	$C_3H_5^+$
0.0321	17.0017	HO^-	0.0295	86.1013	$C_6H_{14}^+$	0.0328	13.0076	CH^-	0.0302	13.0076	CH^-
0.0271	13.0076	CH^-	0.0288	17.0017	HO^-	0.03	25.0112	C_2H^-	0.0278	17.0017	HO^-
0.0251	74.9837	$CH_3SiO_2^-$	0.0239	13.0076	CH^-	0.0239	34.9723	Cl^-	0.0205	77.0348	$C_6H_5^+$
0.0187	149.005	$C_8H_5O_3^-$	0.0232	74.9837	$CH_3SiO_2^-$	0.023	74.9837	$CH_3SiO_2^-$	0.0194	79.0548	$C_6H_7^+$
0.0167	27.9689	Si^+	0.0166	149.005	$C_8H_5O_3^-$	0.017	45.0293	$C_2H_5O^+$	0.0185	91.0513	$C_7H_7^+$
0.0159	222.996	$C_{13}H_3O_4^-$	0.0139	222.996	$C_{13}H_3O_4^-$	0.0169	27.9689	Si^+	0.0175	55.0534	$C_4H_7^+$
0.0151	207.049	$C_{11}H_{11}O_4^+$	0.0131	73.011	C_6H^-	0.0148	75.0477	$C_3H_7O_2^+$	0.0158	39.0224	$C_3H_3^+$

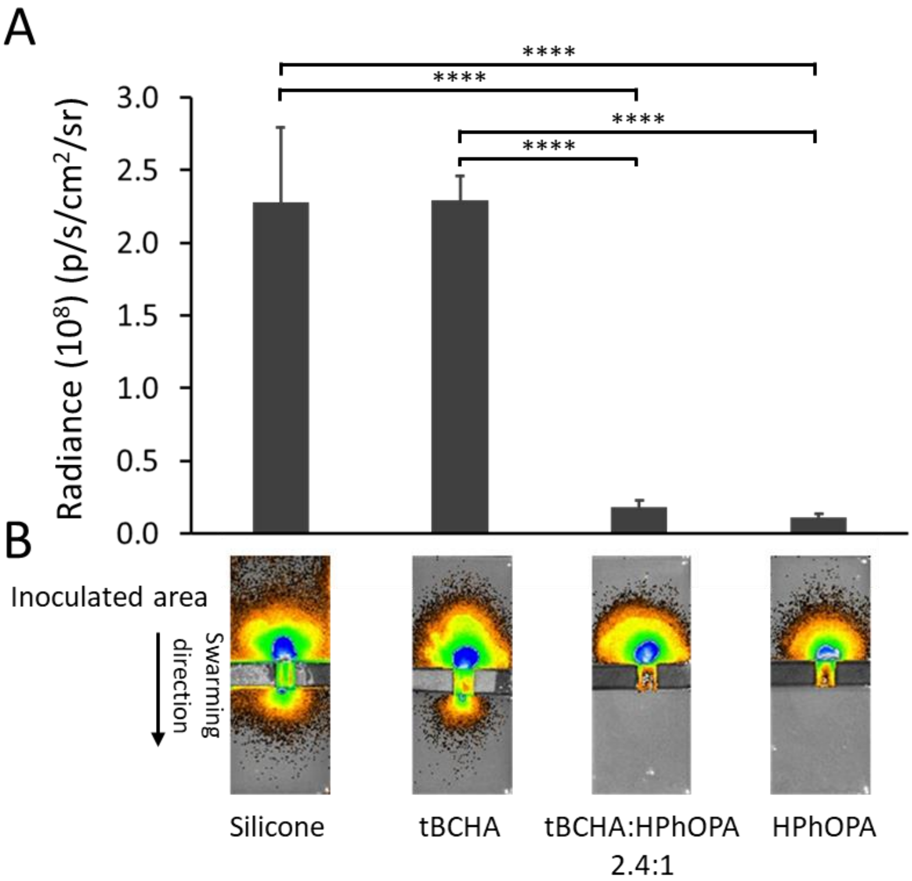

Fig. S5. Swarming motility of *P. mirabilis* 1885 across artificial urine (AU) conditioned silicone catheter bridges coated with tBCHA, HPhOPA or the tBCHA:HPhOPA 2.4:1 copolymer respectively showing (***A***) the fluorescence quantified on the surface of the lower agar block. Values are the mean of three parallel experiments, error bars equal ± one standard deviation for three biological replicates. ****$p<0.0001$. Significance was determined by one-way ANOVA analysis using Tukey's multiple comparisons test. (***B***) Fluorescence images of the agar bridge assembly after 16 h migration across AU-conditioned catheter bridges. Bacteria were inoculated onto the upper agar block and the lower block imaged after 16h.

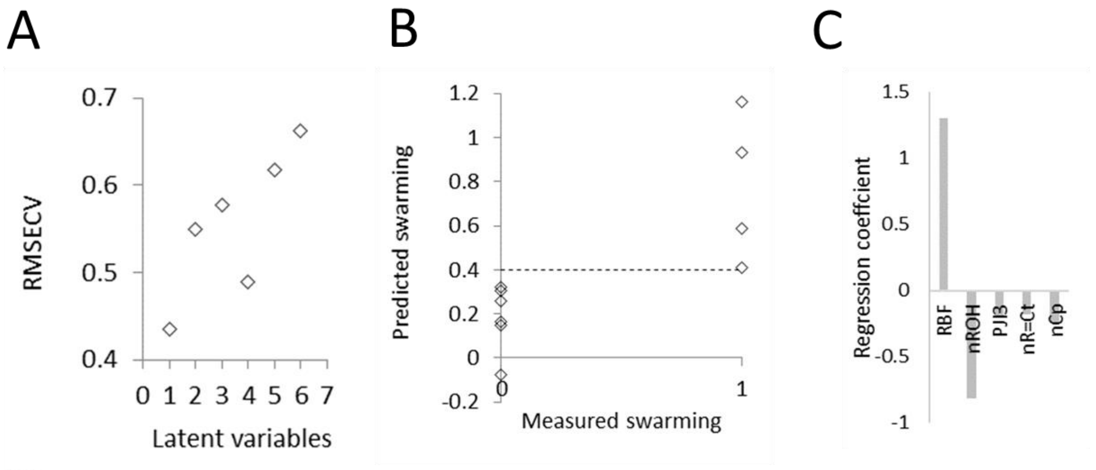

D

Abbreviation	Molecular descriptor description
RBF	rotatable bond fraction
nROH	number of hydroxyl groups
PJI3	3D Petitjean shape index
nR=Ct	number of aliphatic tertiary C(sp2)
nCp	number of terminal primary C(sp3)

Fig. S6 Partial least square (PLS) regression model predicting the ability of a polymer to inhibit swarming from molecular descriptors. (***A***) RMSECV curve for 9 molecular descriptors. (***B***) Measured versus predicted values for the 11 polymers used in this study. A threshold value of 0.4 is shown as a dotted line, above which all polymers able to prevent swarming were successfully predicted. (***C***) The regression coefficient for the final model. (***D***) Abbreviations and names for the molecular descriptors used to form the final model.

Partial least square (PLS) regression model to predict the ability of a polymer to inhibit swarming from molecular descriptors

In the PLS model each of the 4 polymers that resisted swarming had a predicted swarming value greater than 0.4 whilst all of the remaining materials had a predicted swarming value below 0.4, thus, using 0.4 as a threshold value allowed for each of the 11 materials to be correctly assigned as being able to either inhibit or support swarming (**Fig. S6*B***)(32, 45). Each of the molecular descriptors was assigned a regression coefficient from which the influence of a particular descriptor on the ability of a polymer to prevent swarming can be determined by assessing the polarity and magnitude (**Fig. S6*C***). Molecular descriptors associated with molecular rigidity, such as the rotatable bond fraction (RBF) and the 3D Petitjean shape index (PJI3), as well as descriptors associated with hydrophilicity, such as the number of hydroxyl groups (nROH), the number of aliphatic tertiary C(sp2) (nR=Ct) and the number of terminal primary C(sp3) (nCp), were included in the model (**Fig. S6*D***). This suggests that for the 11 polymers studied an interplay of molecular rigidity and hydrophilicity influence the ability of a polymer to inhibit swarming. This is similar to the α parameter that correlated with the ability of polyacrylates with aliphatic carbon pendant groups to prevent bacterial attachment (23). This parameter was derived from the combination of the calculated partition coefficient and the number of rotatable bonds. In the present PLS model the hydrophilicity component is more complicated than for the α parameter, whereby an interplay of both hydrophilic groups (nOH) and hydrophobic groups (nR=Ct and nCP) were required to successfully predict whether a material inhibits swarming.

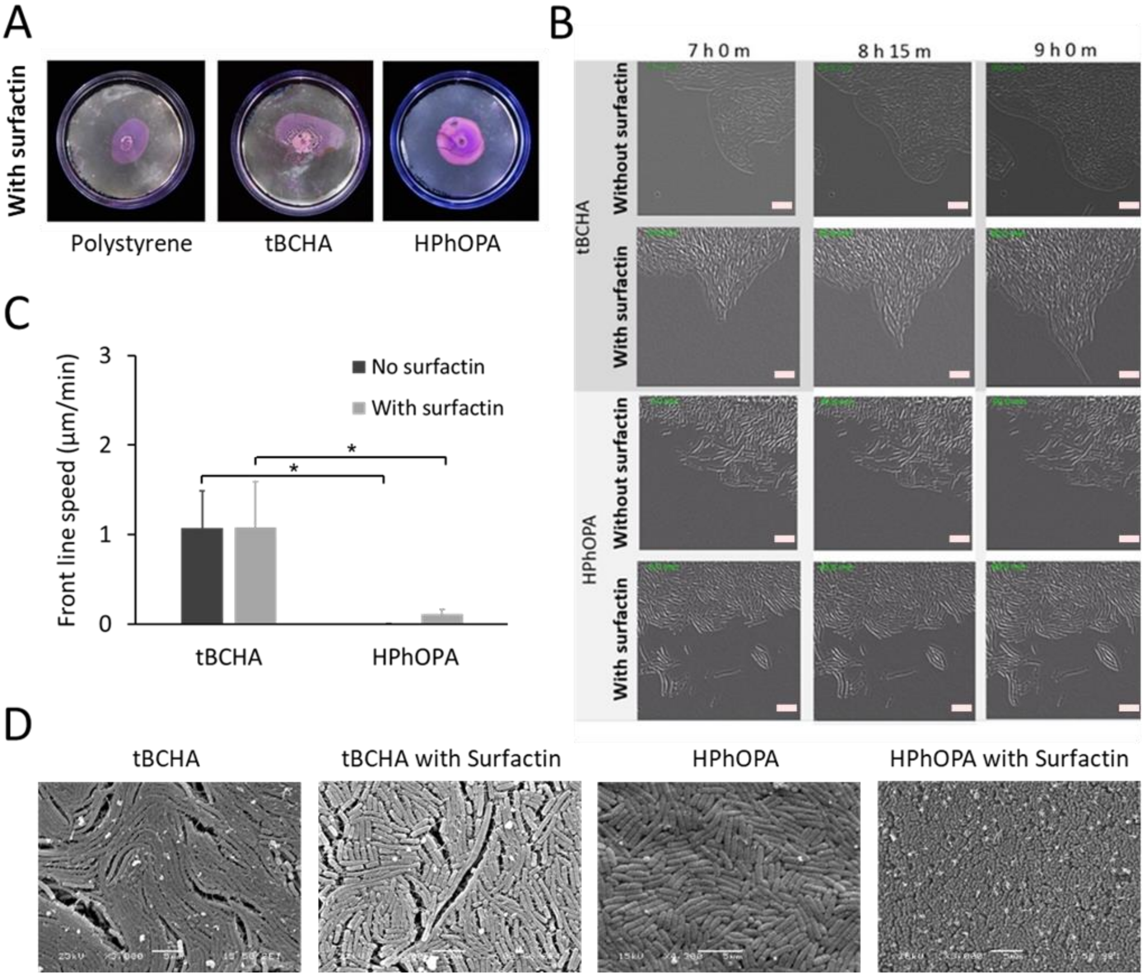

Fig. S7. Impact of surfactin on *P. mirabilis* swarming on poly(HPhOPA). (***A***) Images of crystal violet stained bacteria migrating between agar containing surfactin and, from left to right, uncoated, poly(tBCHA) and poly(HPhOPA) coated polystyrene surfaces. Samples were imaged 7 h after inoculation. (***B***) DIC microscopy time series showing images of *Proteus* cells inoculated onto poly(tBCHA) (top) or poly(HPhOPA) (bottom) and incubated at 37 °C. Images were taken after 7 h, 8.5 and 9 h. Samples without and with 25 µM surfactin are shown. Scale bar, 20 µm. (***C***) Determination of the front line swarming speed on the poly(tBCHA) and poly(HPhOPA) coated surfaces without (dark grey) or with surfactin (light grey). Error bars equal ± one standard deviation for at least three independent replicates. *$p \leq 0.05$. Significance was determined by one-way ANOVA analysis using Tukey's multiple comparisons test. (***D***) Scanning electron microscopy showing the morphology and organisation of *P. mirabilis* cells within the migrating populations on poly(tBCHA) or poly(HPhOPA) with or without surfactin. Scale bars represent 5 µm.

Table S2. Compositional analysis of poly(tBCHA:HPhOPA) by 400 MHz ^{1}H-NMR and chloroform gel permeation chromatography. Molar ratios shown.

Monomer Feed Ratio (tBCHA:HPhOPA)	Polymer composition ratio (tBCHA:HPhOPA)	Number average molecular weight kDa (Mn)	Polydispersity index (PDI)
2.1:3.0	0.9:3.0	118	3.3
3.0:2.0	2.4:3.0	105	6.1
4.0:1.0	2.4:1.0	126	6.3

Table S3. Compositional analysis of scaled up poly(tBCHA:HPhOPA) by 400 MHz ^{1}H-NMR. Molar ratios shown.

Monomer Feed Ratio (tBCHA:HPhOPA)	Polymer composition (tBCHA:HPhOPA)	Scaled up polymers (tBCHA:HPhOPA)	tBCHA % difference
3.0:2.0	2.4:3.0	1.8:2.0	+ 11.1
4.0:1.0	2.4:1.0	4.1:2.0	- 9.8

Table S4 Patient Information relating to urine samples collected pre- and post- catheterization.

Number of Patients	Number of Urine Samples		Mean Indwelling Time (Days)	Range of Indwelling Times (Days)	Mean Patient Age (Years)	Patient Gender	
	Pre	Post				Male	Female
8	8	8	2.5	1-6	51	7	1

A

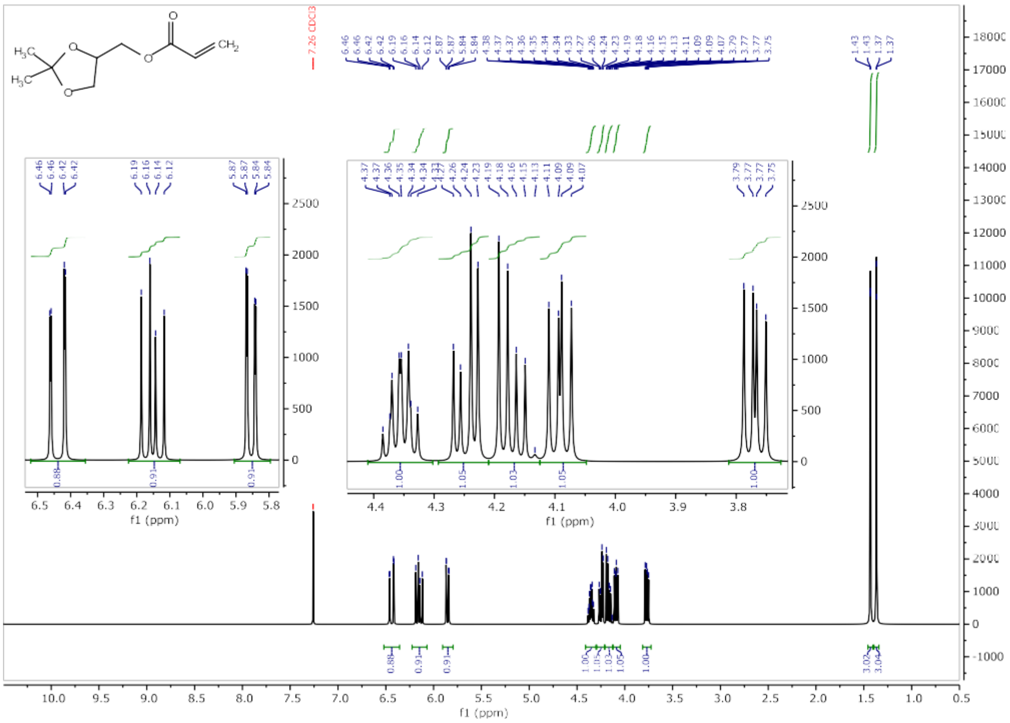

B

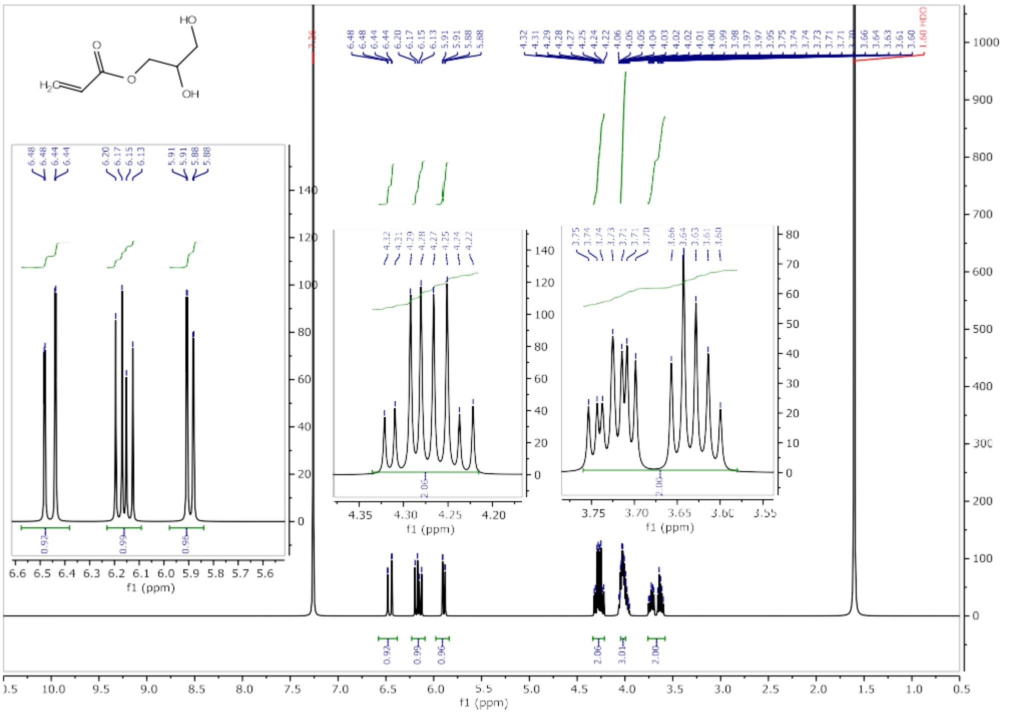

Fig. S8. H-NMR analysis of synthesized monomers (*A*) DMDA and (*B*) DHPA. (*A*) ^{1}H NMR (400 MHz,) δ 6.44 (dd, *J* = 17.3, 1.4 Hz, 1H), 6.15 (dd, *J* = 17.3, 10.4 Hz, 1H), 5.85 (dd, *J* = 10.4, 1.4 Hz, 1H), 4.37 – 4.33 (m, 1H), 4.25 (dd, *J* = 11.5, 4.7 Hz, 1H), 4.17 (dd, *J* = 11.5, 6.0 Hz, 1H), 4.09 (dd, *J* = 8.5, 6.4 Hz, 1H), 3.77 (dd, *J* = 8.5, 6.0 Hz, 1H), 1.43 – 1.43 (m, 3H), 1.37 – 1.37 (m, 3H). (*B*) ^{1}H NMR (400 MHz,) δ 6.46 (dd, *J* = 17.3, 1.3 Hz, 1H), 6.16 (dd, *J* = 17.3, 10.4 Hz, 1H), 5.89 (dd, *J* = 10.4, 1.3 Hz, 1H), 4.27 (ddd, *J* = 11.7, 11.7, 5.4 Hz, 2H), 4.06 – 3.95 (m, 3H), 3.76 – 3.58 (m, 2H).

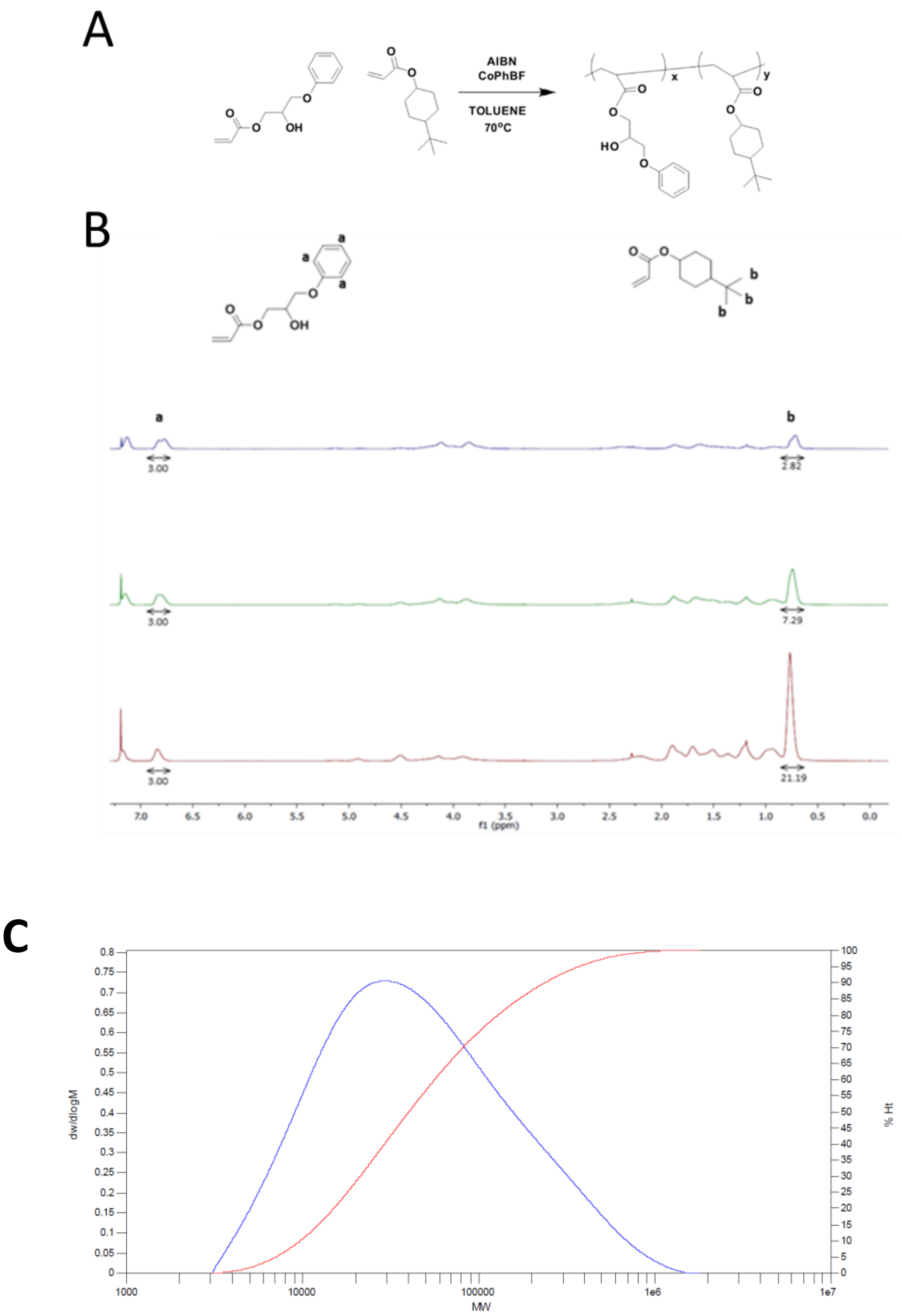

Fig. S9. (***A***) Synthesis of poly(tert-butylcyclohexylacrylate(tBCHA)-co-hydroxy-3-phenoxypropyl acrylate (HPhOPA)). (***B***) Stacked proton NMR spectra of tBCHA:HPhOPA copolymers with 0.9:3 (blue), 2.4:3 (green) and 2.4:1 (red) monomer ratios. Chemical structures of HPhOPA (left) and tBCHA (right) are also shown indicating protons associated with the benzyl ring (**A**) or tert-butyl groups (***B***) that were used to quantify the monomer content from the NMR spectra. (***C***) GPC analysis of copolymer tBCHA:HPhOPA 2.4:1 showing the differential (blue) and cumulative (red) molar mass distributions.

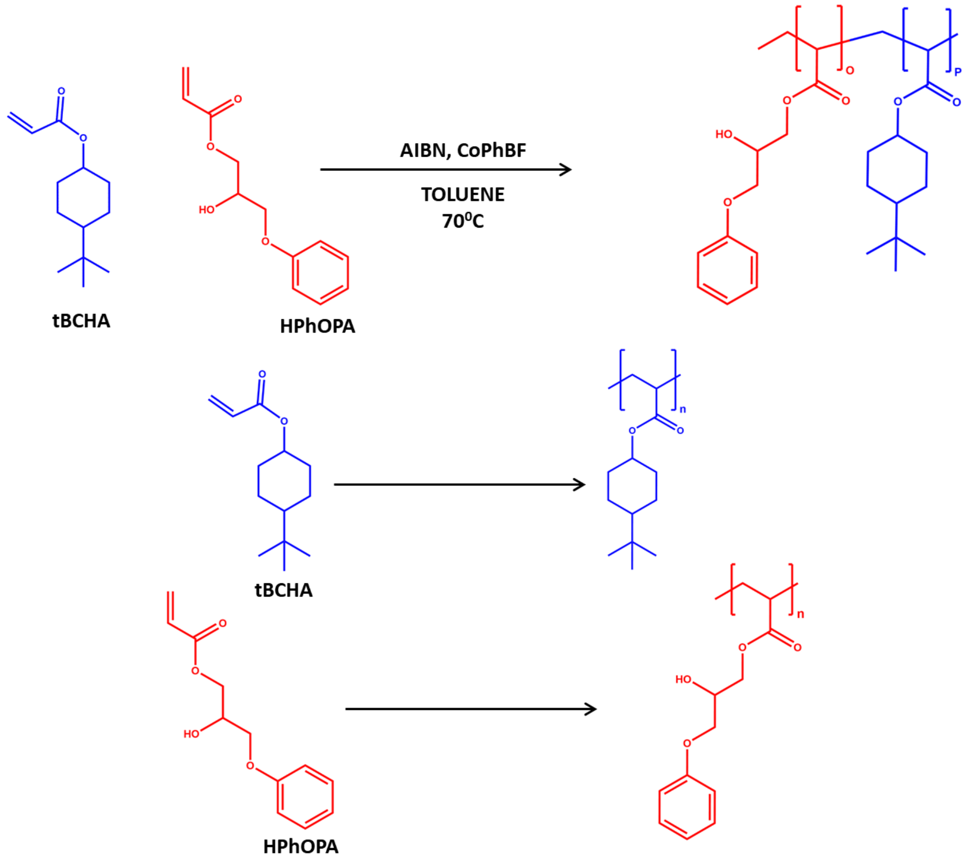

Fig. S10. Structures of tBCHA, HPhoPA and their crosslinked homo- and hetero- polymers

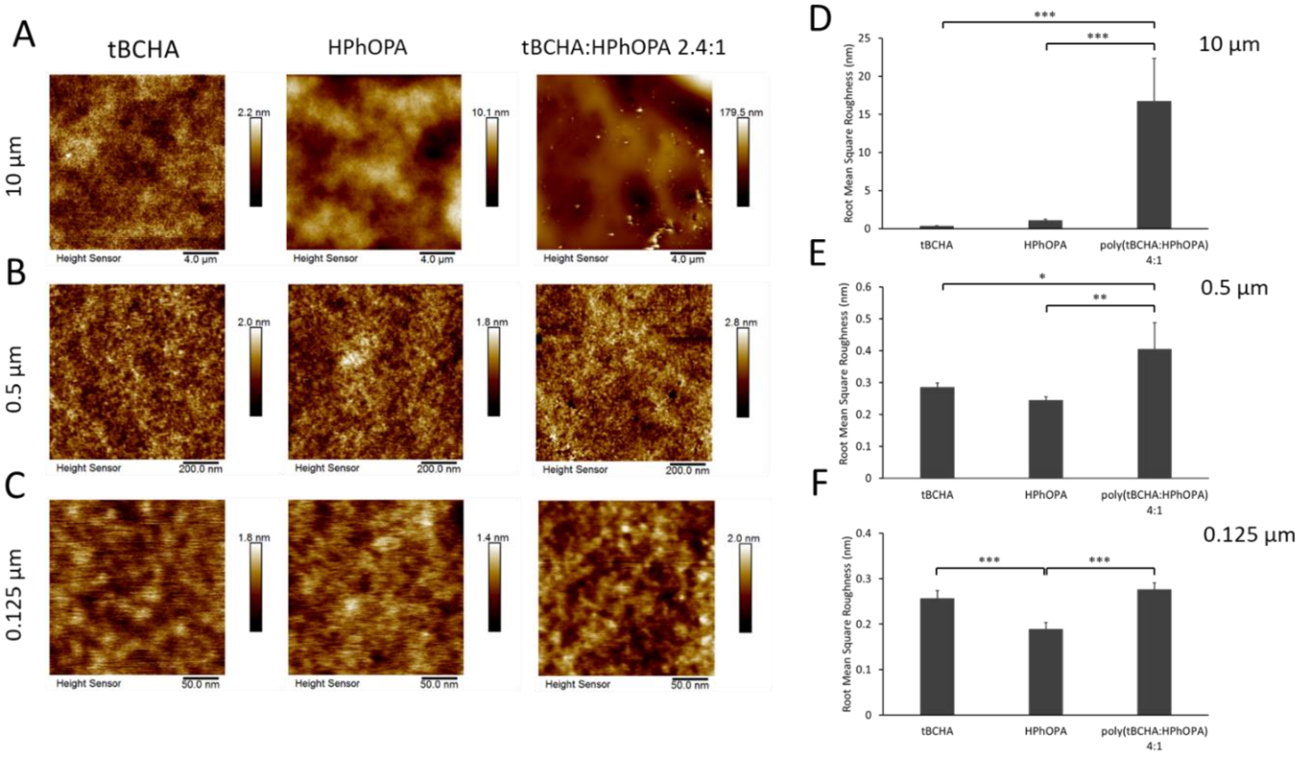

Fig. S11. Surface roughness of the HPhOPA and tBCHA homopolymers and the tBCHA:HPhOPA 2.4:1 copolymer coatings determined using atomic force microscopy (AFM). (***A-C***) the polymer surface imaged at 10, 0.5 and 0.125 µm from the surface. (***D-F***). Determination of the root mean square roughness (nm) of the homopolymers tBCHA and HPhOPA, and the co-polymer tBCHA:HPhOPA 2.4:1 copolymer imaged at 10 µm, 0.5 µm and 0.125 µm from the surface. Error bars equal ± one standard deviation for four biological replicates. *$p \leq 0.05$; **$p \leq 0.01$; ***$p \leq 0.001$. Significance was determined by one-way Anova analysis using Tukey's multiple comparisons test.

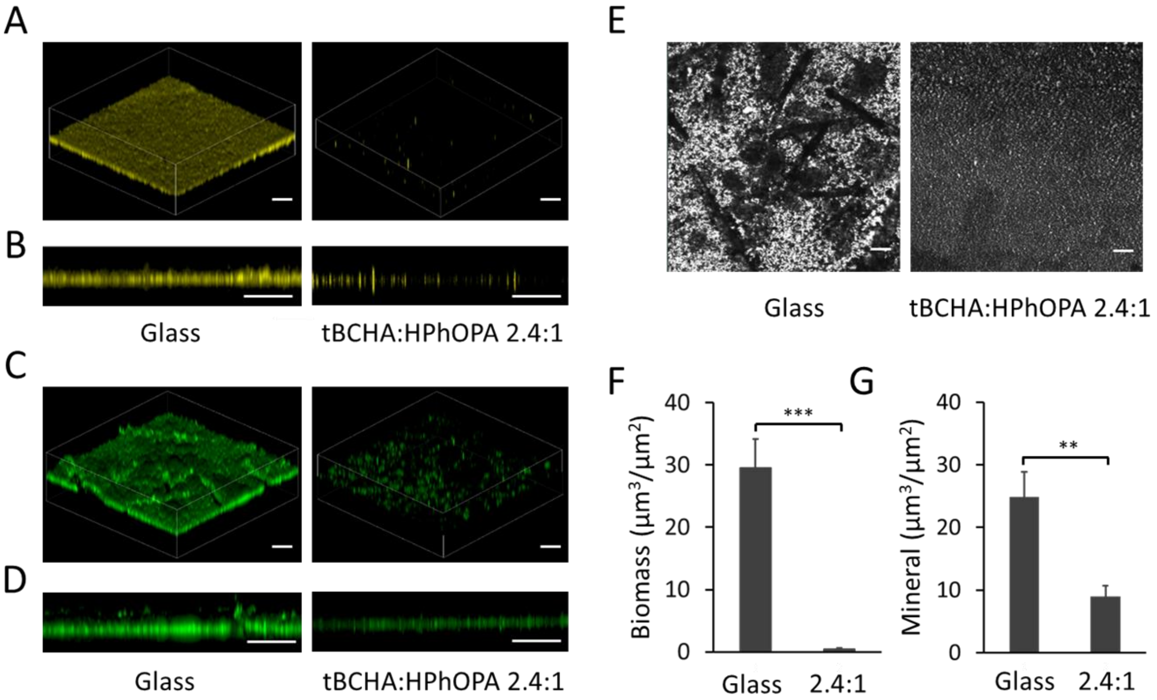

Fig. S12. *Proteus* biofilm formation and biomineralization on the tBCHA:HPhOPA 2.4:1 copolymer. GFP-labelled *Proteus* was cultured on glass or copolymer coated coverslips for 72 h in AU. Biofilm formation (***A*** and ***B***) and biomineralization detected by calcein staining (***C*** and ***D***) were imaged by confocal microscopy. (***E***) Bright-field images showing *Proteus*-dependent biomineralization. Scale bars, 50µm. Quantification of biomass (***F***) and biomineralization (***G***) by *Proteus* on glass or on copolymer coated coverslips. Values given are the means of five independent replicates. Error bars are ± one standard deviation unit. **$p \leq 0.01$; ***$p \leq 0.005$. Significance was determined by unpaired Student *t*-test.

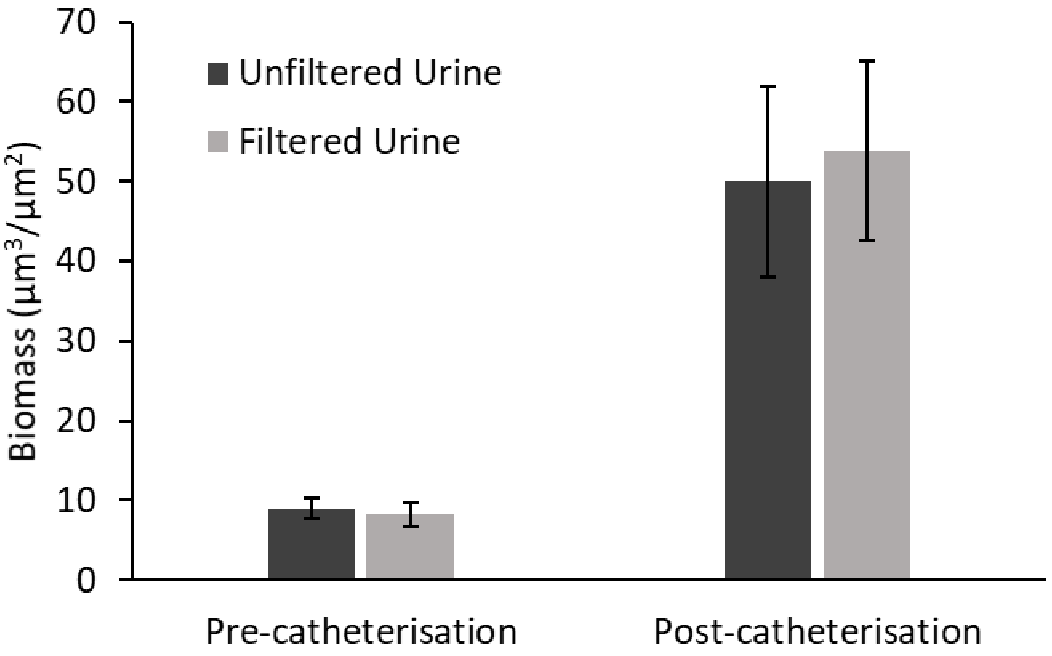

Fig. S13 Filtration by sterilization did not affect biofilm development on catheter sections. *S. aureus (gfp*-tagged*)* was grown on silicone catheter sections pre-conditioned with urine taken from a patient pre- and post-catheterization respectively. Urine samples were either not filtered or filtered with 0.22µm membrane filter (Millipore). Biofilm biomass shows no significant difference between unfiltered and filtered urine. Error bars are ±SD.

A

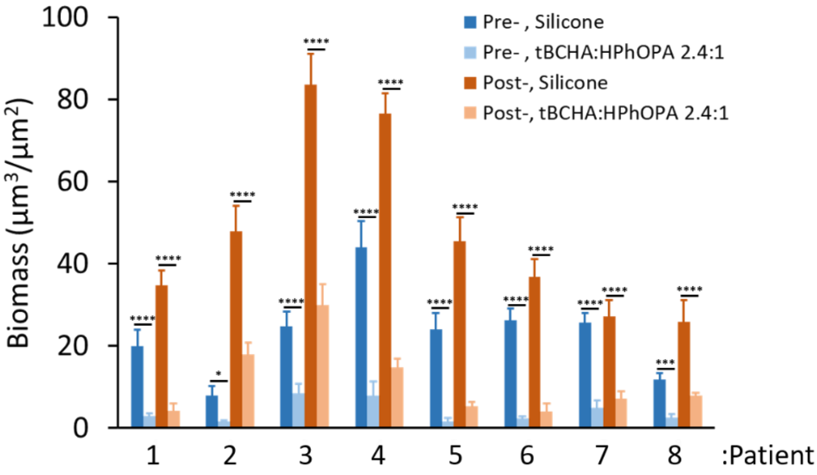

B

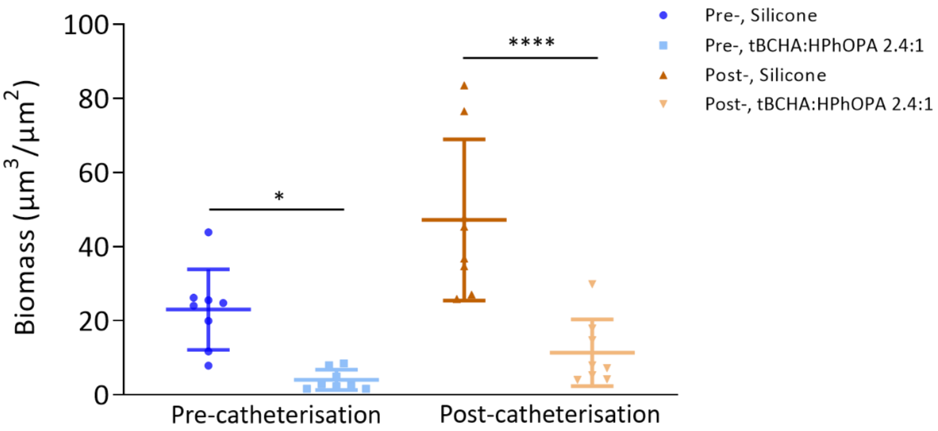

Fig. S14. (***A***) Biofilm biomass formed by GFP-tagged *S. aureus* on silicone (dark blue & dark brown) or poly(tBCHA:HPhOPA) (light blue & light brown) catheter segments conditioned with pre- (blue) or post- (brown) catheterisation urine from 8 patients. (***B***) Scatter plot showing the overall mean for *S.aureus* biomass. *$p \leq 0.05$; **$p \leq 0.01$; *** $p \leq 0.001$; ****$p \leq 0.0001$. Significance was determined by two-way Anova analysis using Sidak's multiple comparisons test.

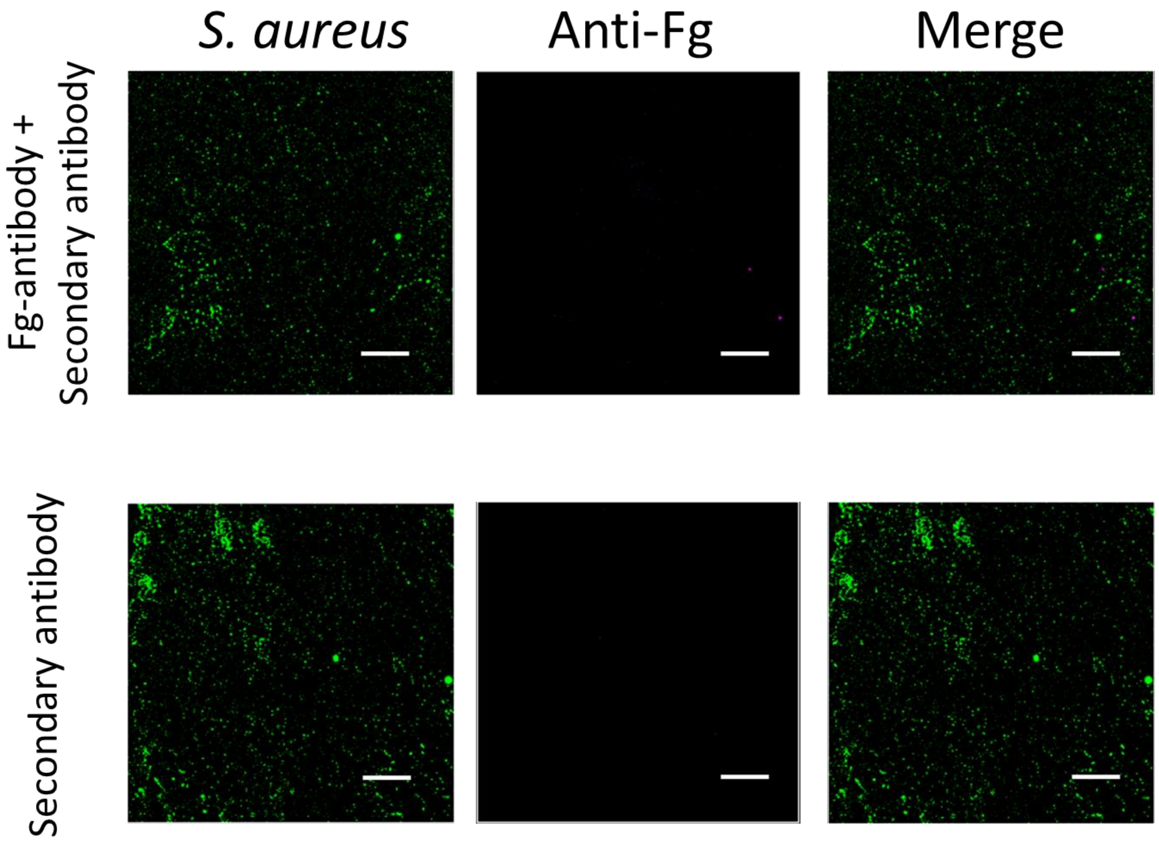

Fig. S15. *S. aureus* (*gfp*-labelled) biofilm on silicone segments were probed with primary (rabbit antibody to human fibrinogen) and/or secondary anti-rabbit Qdot700 conjugate. No cross reactivity with the bacterial cells was observed. Scale bars, 50 μM.

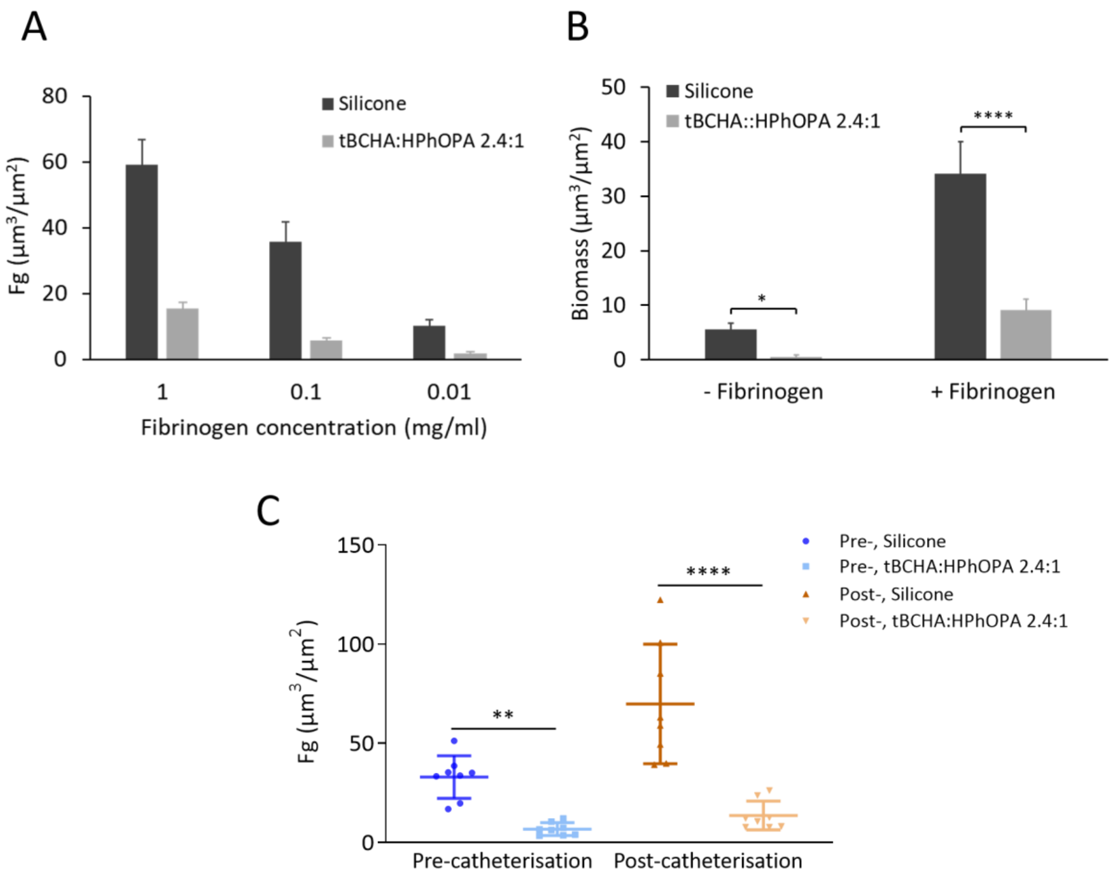

Fig. S16 (***A***) Human fibrinogen surface coverage on silicone and poly(tBCHA:HPhOPA) *in vitro* following pre-conditioning with a range of fibrinogen concentrations. (***B***) Quantification of *S. aureus* biomass on silicone and copolymer after pre-conditioning with Fg (0.1 mg/ml). (***C***) Scatter plot showing the mean Fg concentration on silicone and poly(tBCHA:HPhOPA after surface conditioning with pre and post-catheterization urine from 8 patients. Values given are the means of five images, error bars are ± one standard deviation unit. **p*≤0.05; ***p*≤0.01; *** *p*≤0.001; *****p*≤0.0001. Significance was determined by two-way Anova analysis using Sidak's multiple comparisons test.

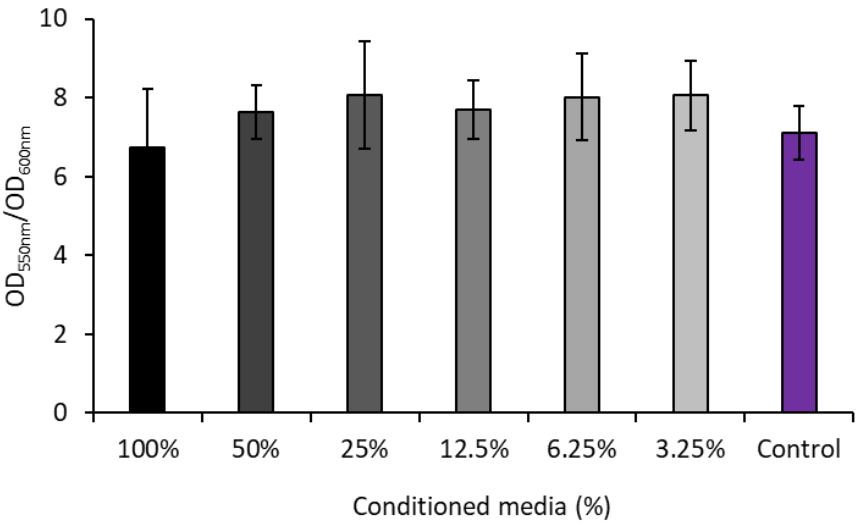

Fig. S17. MTT cytotoxicity assay. Conditioned media were generated by incubation of poly(tBCHA:HPhOPA) coated coverslips in tissue culture media (MEM) for 8 days to allow leaching of soluble products. MRC-5 fibroblasts in 96-well plates were incubated in a range of dilutions of conditioned media or in unconditioned media. MTT assays were carried out for 72h at 37°C in 5% CO_2, A_{550} was quantified and the data normalised to cell density. Values given are the means of seven independent replicates. Error bars are ± one standard deviation unit. No reduction in cell viability with the MTT assay was observed.

Videos S1 and S2.

DIC videos of the migrating front of *P. mirabilis* 1885 swarming on a coating of tBCHA (**video S1**) or HPhOPA (**video S2**) showing the elongation and alignment of cells at the moving front on tBCHA and the absence of this cell organisation on HPhOPA.

REFERENCES

1. R. O. Darouiche, Device-associated infections: A macroproblem that starts with microadherence. *Clin. Infect. Dis.* **33**, 1567–1572 (2001).

2. C. S. Hollenbeak, A. L. Schilling, The attributable cost of catheter-associated urinary tract infections in the United States: A systematic review. *Am. J. Infect. Control* **46**, 751–757 (2018).

3. E. C. Bursle, J. Dyer, D. F. M. Looke, D. A. J. McDougall, D. L. Paterson, E. G. Playford Risk factors for urinary catheter associated bloodstream infection. *J. Infect.* **70**, 585–591 (2015).

4. B. Foxman, The epidemiology of urinary tract infection. *Nat. Rev. Urol.* **7**, 653–660 (2010).

5. C. E. Armbruster, S. N. Smith, A. O. Johnson, V. De Ornellas, K. A. Eaton, A. Yep, L. Mody, W. Wu, H. L. T. Mobley, The pathogenic potential of *Proteus mirabilis* is enhanced by other uropathogens during polymicrobial urinary tract infection. *Infect. Immun.* **85**, e00808-16 (2017).

6. S. M. Jacobsen, M. E. Shirtliff, *Proteus mirabilis* biofilms and catheter-associated urinary tract infections. *Virulence* **2**, 460–465 (2011).

7. T. M. Hooton, S. F. Bradley, D. D. Cardenas, R. Colgan, S. E. Geerlings, J. C. Rice, S. Saint, A. J. Schaeffer, P. A. Tambayh, P. Tenke, L. E. Nicolle; Infectious Diseases Society of America Diagnosis, prevention, and treatment of catheter-associated urinary tract infection in adults: 2009 International Clinical Practice Guidelines from the Infectious Diseases Society of America. *Clin. Infect. Dis.* **50**, 625–663 (2010).

8. L. E. Nicolle, Catheter associated urinary tract infections. *Antimicrob. Resist. Infect. Control* **3**, 23 (2014).

9. P. Singha, J. Locklin, H. Handa, A review of the recent advances in antimicrobial coatings for urinary catheters. *Acta Biomater.* **50**, 20–40 (2017).

10. L. E. Fisher, A. L. Hook, W. Ashraf, A. Yousef, D. A. Barrett, D. J. Scurr, X. Chen, E. F. Smith, M. Fay, C. D. J. Parmenter, R. Parkinson, R. Bayston, Biomaterial modification of urinary catheters with antimicrobials to give long-term broadspectrum antibiofilm activity. *J. Control. Release* **202**, 57–64 (2015).

11. K. Schumm, T. B. Lam, Types of urethral catheters for management of short-term voiding problems in hospitalised adults. *Cochrane Database Syst. Rev.* **16**, CD004013 (2008).

12. K. K. Kumarasamy, M. A. Toleman, T. R. Walsh, J. Bagaria, F. Butt, R. Balakrishnan, U. Chaudhary, M. Doumith, C. G. Giske, S. Irfan, P. Krishnan, A. V. Kumar, S. Maharjan, S. Mushtaq, T. Noorie, D. L. Paterson, A. Pearson, C. Perry, R. Pike, B. Rao, U. Ray, J. B. Sarma, M. Sharma, E. Sheridan, M. A. Thirunarayan, J. Turton, S. Upadhyay, M. Warner, W. Welfare, D. M. Livermore, N. Woodford, Emergence of a new antibiotic resistance mechanism in India, Pakistan, and the UK: A molecular, biological, and epidemiological study. *Lancet Infect. Dis.* **10**, 597–602 (2010).

13. R. J. Broomfield, S. D. Morgan, A. Khan, D. J. Stickler Crystalline bacterial biofilm formation on urinary catheters by urease-producing urinary tract pathogens: A simple method of control. *J. Med. Microbiol.* **58**, 1367–1375 (2009).

14. A. N. Norsworthy, M. M. Pearson, From catheter to kidney stone: The uropathogenic lifestyle of *Proteus mirabilis*. *Trends Microbiol.* **25**, 304–315 (2017).

15. S. M. Jacobsen, D. J. Stickler, H. L. T. Mobley, M. E. Shirtliff, Complicated catheter-associated urinary tract infections due to *Escherichia coli* and *Proteus mirabilis. Clin. Microbiol. Rev.* **21**, 26–59 (2008).

16. N. S. Morris, D. J. Stickler, C. Winters, Which indwelling urethral catheters resist encrustation by *Proteus mirabilis* biofilms? *Br. J. Urol.* **80**, 58–63 (1997).

17. N. Sabbuba, G. Hughes, D. J. Stickler, The migration of *Proteus mirabilis* and other urinary tract pathogens over Foley catheters. *BJU Int.* **89**, 55–60 (2002).

18. A. L. Flores-Mireles, J. N. Walker, T. M. Bauman, A. M. Potretzke, H. L. Schreiber IV, A. M. Park, J. S. Pinkner, M. G. Caparon, S. J. Hultgren, A. Desai, Fibrinogen release and deposition on urinary catheters placed during urological procedures. *J. Urol.* **196**, 416–421 (2016).

19. J. N. Walker, A. L. Flores-Mireles, C. L. Pinkner, H. L. Schreiber IV, M. S. Joens, A. M. Park, A. M. Potretzke, T. M. Bauman, J. S. Pinkner, J. A. J. Fitzpatrick, A. Desai, M. G. Caparon, S. J. Hultgren, Catheterization alters bladder ecology to potentiate *Staphylococcus aureus* infection of the urinary tract. *Proc. Natl. Acad. Sci. U.S.A.* **114**, E8721–E8730 (2017).

20. A. L. Hook, D. G. Anderson, R. Langer, P. Williams, M. C. Davies, M. R. Alexander, High throughput methods applied in biomaterial development and discovery. *Biomaterials* **31**, 187–198 (2010).

21. A. L. Hook, C. Y. Chang, J. Yang, S. Atkinson, R. Langer, D. G. Anderson, M. C. Davies, P. Williams, M. R. Alexander, Discovery of novel materials with broad resistance to bacterial attachment using combinatorial polymer microarrays. *Adv. Mater.* **25**, 2542–2547 (2013).

22. A. L. Hook, C. Y. Chang, J. Yang, J. Luckett, A. Cockayne, S. Atkinson, Y. Mei, R. Bayston, D. J. Irvine, R. Langer, D. G. Anderson, P. Williams, M. C. Davies, M. R. Alexander, Combinatorial discovery of polymers resistant to bacterial attachment. *Nat. Biotechnol.* **30**, 868–875 (2012).

23. O. Sanni, C. Y. Chang, D. G. Anderson, R. Langer, M. C. Davies, P. M. Williams, P. Williams, M. R. Alexander, A. L. Hook, Bacterial attachment to polymeric materials correlates with molecular flexibility and hydrophilicity. *Adv. Healthc. Mater.* **4**, 695–701 (2015).

24. A. A. Dundas, O. Sanni, J.-F. Dubern, G. Dimitrakis, A. L. Hook, D. J. Irvine, P. Williams, M. R. Alexander, Validating a predictive structure–property relationship by discovery of novel polymers which reduce bacterial biofilm formation. *Adv. Mater.* **31**, 1903513 (2019).

25. P. Mikulskis, A. Hook, A. A. Dundas, D. Irvine, O. Sanni, D. Anderson, R. Langer, M. R. Alexander, P. Williams, D. A. Winkler, Prediction of broad-spectrum pathogen attachment to coating materials for biomedical devices. *ACS Appl. Mater. Interfaces* **10**, 139–149 (2018).

26. M. R. Alexander, P. Williams, Water contact angle is not a good predictor of biological responses to materials. *Biointerphases* **12**, 02C201 (2017).

27. K. Adlington, N. T. Nguyen, E. Eaves, J. Yang, C. Y. Chang, J. Li, A. L. Gower, A. Stimpson, D. G. Anderson, R. Langer, M. C. Davies, A. L. Hook, P. Williams, M. R. Alexander, D. J. Irvine, Application of targeted molecular and material property optimization to bacterial attachment-resistant (meth)acrylate polymers. *Biomacromolecules* **17**, 2830–2838 (2016).

28. R. M. Harshey, J. D. Partridge, Shelter in a swarm. *J. Mol. Biol.* **427**, 3683–3694 (2015).

29. D. J. Stickler, Clinical complications of urinary catheters caused by crystalline biofilms: Something needs to be done. *J. Intern. Med.* **276**, 120–129 (2014).

30. A. L. Hook, D. J. Scurr, ToF-SIMS analysis of a polymer microarray composed of poly(meth)acrylates with C6 derivative pendant groups. *Surf. Interface Anal.* **48**, 226–236 (2016).

31. A. D. Celiz, H. C. Harrington, A. L. Hook,, High throughput assessment and chemometric analysis of the interaction of epithelial and fibroblast cells with a polymer library. *Appl. Surf. Sci.* **313**, 926–935 (2014).

32. D. A. Winkler, F. R. Burden, Robust, quantitative tools for modelling ex-vivo expansion of haematopoietic stem cells and progenitors. *Mol. Biosyst.* **8**, 913–920 (2012).

33. F. D. Williams, R. H. Schwarzhoff, Nature of the swarming phenomenon in *Proteus*. *Annu. Rev. Microbiol.* **32**, 101–138 (1978).

34. D. B. Kearns, A field guide to bacterial swarming motility. *Nat. Rev. Microbiol.* **8**, 634–644 (2010).

35. C. E. Armbruster, S. N. Smith, A. Yep, H. L. T. Mobley,, Increased incidence of urolithiasis and bacteremia during *Proteus mirabilis* and *Providencia stuartii* coinfection due to synergistic induction of urease activity. *J Infect Dis* **209**, 1524–1532 (2014).

36. C. E. Armbruster, H. L. T. Mobley, Merging mythology and morphology: The multifaceted lifestyle of *Proteus mirabilis*. *Nat. Rev. Microbiol.* **10**, 743–754 (2012).

37. D. Gygi, M. M. Rahman, H. C. Lai, R. Carlson, J. Guard-Petter, C. Hughes, A cell-surface polysaccharide that facilitates rapid population migration by differentiated swarm cells of *Proteus mirabilis*. *Mol. Microbiol.* **17**, 1167–1175 (1995).

38. X. Li, N. Lu, H. R. Brady, A. I. Packman, Biomineralization strongly modulates the formation of *Proteus mirabilis* and *Pseudomonas aeruginosa* dual-species biofilms. *FEMS Microbiol. Ecol.* **92**, (2016).

39. T. Brooks, C. W. Keevil, A simple artificial urine for the growth of urinary pathogens. *Lett. Appl. Microbiol.* **24**, 203–206 (1997).

40. B. Koch, L. E. Jensen, O. Nybroe, A panel of Tn7-based vectors for insertion of the *gfp* marker gene or for delivery of cloned DNA into Gram-negative bacteria at a neutral chromosomal site. *J. Microbiol. Methods* **45**, 187–195 (2001).

41. T. K. Prajsnar, R. Hamilton, J. Garcia-Lara, G. McVicker, A. Williams, M. Boots, S. J. Foster, S. A. Renshaw, A privileged intraphagocyte niche is responsible for disseminated infection of *Staphylococcus aureus* in a zebrafish model. *Cell. Microbiol.* **14**, 1600–1619 (2012).

42. R. Popat, S. A. Crusz, M. Messina, P. Williams, S. A. West, S. P. Diggle, Quorum-sensing and cheating in bacterial biofilms. *Proc. R. Soc. B Biol. Sci.* **279**, 4765–4771 (2012).

43. A. Shrinidhi, Diels-Alder reaction with hydrophilic dienes and dienophiles. *Chemistryselect* **1**, 3016–3021 (2016).

44. B. J. Tyler, A. Hook, A. Pelster, P. Williams, M. Alexander, H. F. Arlinghaus, Development and characterization of a stable adhesive bond between a poly(dimethylsiloxane) catheter material and a bacterial biofilm resistant acrylate polymer coating. *Biointerphases* **12**, 02C412 (2017).

45. T. Mossmann, Rapid colorimetric assay for cellular growth and survival: Application to proliferation and cytotoxicity assays. *J. Immunol. Methods* **65**, 55–63 (1983).

46. A. L. Hook, D. J. Scurr, J. C. Burley, R. Langer, D. G. Anderson, M. C. Davies, M. R. Alexander, Analysis and prediction of defects in UV photo-initiated polymer microarrays. *J. Mater. Chem. B*, **1**, 1035–1043 (2013).
